# Dynamical refinement with multipolar electron scattering factors

**DOI:** 10.1107/S2052252524001763

**Published:** 2024-03-21

**Authors:** Barbara Olech, Petr Brázda, Lukas Palatinus, Paulina Maria Dominiak

**Affiliations:** a Biological and Chemical Research Centre, Department of Chemistry, University of Warsaw, Warsaw, Poland; b Institute of Physics of the Czech Academy of Sciences, Na Slovance 1999/2, 182 00 Prague, Czechia; Istituto Italiano di Tecnologia, Italy

**Keywords:** electron crystallography, 3D electron diffraction, microcrystal electron diffraction, dynamical refinement, transferable aspherical atom model, multipolar scattering factors, quantum crystallography

## Abstract

Dynamical refinement is a well established method for refining crystal structures against 3D electron diffraction (ED) data and its benefits have been discussed in the literature [Palatinus, Petříček & Corrêa, (2015). *Acta Cryst.* A**71**, 235–244; Palatinus, Corrêa *et al.* (2015). *Acta Cryst.* B**71**, 740–751]. However, until now, dynamical refinements have only been conducted using the independent atom model (IAM). Recent research has shown that a more accurate description can be achieved by applying the transferable aspherical atom model (TAAM), but this has been limited only to kinematical refinements [Gruza *et al.* (2020). *Acta Cryst.* A**76**, 92–109; Jha *et al.* (2021). *J. Appl. Cryst.* **54**, 1234–1243]. In this study, we combine dynamical refinement with TAAM for the crystal structure of 1-methyl­uracil, using data from precession ED. Our results show that this approach improves the residual Fourier electrostatic potential and refinement figures of merit. Furthermore, it leads to systematic changes in the atomic displacement parameters of all atoms and the positions of hydrogen atoms. We found that the refinement results are sensitive to the parameters used in the TAAM modelling process. Though our results show that TAAM offers superior performance compared with IAM in all cases, they also show that TAAM parameters obtained by periodic DFT calculations on the refined structure are superior to the TAAM parameters from the UBDB/MATTS database. It appears that multipolar parameters transferred from the database may not be sufficiently accurate to provide a satisfactory description of all details of the electrostatic potential probed by the 3D ED experiment.

## Abstract 2

Transferable aspherical atom model dynamical refinement on precession electron diffraction data for 1-methyl­uracil crystals offers superior performance compared with the independent atom model and reveals that the quality of 3D electron diffraction data and dynamical refinement is already sufficient to detect minute variations of the electrostatic potential caused by bonding and intermolecular interactions.

## 1. Introduction

Fine details about redistribution of electron densities due to chemical bonds and intermolecular interactions are essential for understanding the functionality of small molecules and the crystalline materials they constitute. It is believed that bonding effects should be easily visible in electron diffraction (ED) data, especially for materials comprised of elements with low atomic numbers. ED methods have made enormous progress in recent years. There are available structures from 3D ED data of atomic and near-atomic resolution (Gemmi *et al.*, 2019 ▸). The possibility to refine not only atomic positions, but also to observe accurate features of the electrostatic potential and underlying electron density, is within our reach. However, to verify conclusions based on observations, quantum crystallography methods must be applied. Also, the knowledge gained from X-ray diffraction (XRD) experiments would be beneficial.

Interest in electrostatic potential observed in ED experiments reaches as far back as the end of 20th century. In the work by Zuo *et al.* (1999 ▸), the charge-density distribution of Cu_2_O was mapped using convergent-beam electron diffraction (CBED) combined with X-ray diffraction. In the work by Tsirelson *et al.* (2001 ▸), experimental electrostatic potential maps of rock-salt crystals were studied. The bonding electrostatic potential of the aluminium crystal structure was investigated by quantitative CBED by Nakashima (2011 ▸, 2017 ▸). Also, studies based on electron diffraction structure analysis (EDSA) were carried out to analyse electrostatic potential. Reflections were measured on polycrystalline samples assuming that their intensities in majority are well described within the kinematical theory. Multipolar model at various levels of expansion can be refined directly on kinematical electron structure factors. Exemplary studies on electrostatic potential distributions were performed for high-symmetry inorganic structures (Avilov, 2003 ▸; Novikova *et al.*, 2018 ▸). For organic samples, the only (to the best of our knowledge) well documented charge density refinement on ED data was published by Wu & Spence (2003 ▸). The authors used selected low-angle reflections to perform dynamical refinement of a mid-bond charge-cloud ionic model to obtain deformation charge density maps and partial charges on atoms in crystals of copper phthalocyanine.

For XRD, it is possible to experimentally, qualitatively and quantitatively reach subatomic features of electron density. By using sophisticated models, the distribution of electron density can be refined. There are two main approaches in the field: multipole model and wavefunction based methods. Among the first type, the most common is the Hansen–Coppens multipole model (Hansen & Coppens, 1978 ▸). It requires high-quality and sub-atomic resolution data. There are numerous papers presenting experimental charge density studies (Tolborg & Iversen, 2019 ▸). The refinable parameters are atomic positions, atomic displacement parameters, populations of spherical harmonics and parameters of contraction–expansion, thus a model of both the atomic and the electronic structure of the crystal is obtained from experimental data.

For data of resolution worse than *d*_min_ = 0.5 Å, there are insufficient data points to refine all multipole model parameters. Therefore, pre-determined parameters of populations of spherical harmonics and parameters of contraction–expansion can be transferred onto the crystal structure under study and constrained, and during the refinement only the atomic model of the crystal structure is refined (atomic positions and atomic displacement parameters). Such approach is named the transferable aspherical atom model (TAAM) refinement. Usually, parameters for TAAM are taken from a databank. There are several databanks that offer such parameters: UBDB/MATTS (Jarzembska & Dominiak, 2012 ▸; Dominiak *et al.*, 2007 ▸; Jha *et al.*, 2022 ▸), ELMAM2 (Domagała & Jelsch, 2008 ▸) and INVARIOM (Dittrich *et al.*, 2004 ▸). To build the UBDB/MATTS databank the experimental geometries of many molecules are taken, and multipole model parameters are refined against theoretically computed structure factors for these molecules in the gas phase. Next, the parameters are averaged over hundreds of atoms of similar chemical topology. For organic molecules, TAAM refinement is always possible when the independent atom model (IAM) is available. Thanks to the databank approach and analytical (multipolar) representation of scattering factors, TAAM refinement is almost as fast as IAM refinement. In the case of XRD, TAAM results in a better fit of the model to the experimental data and a more accurate crystal atomic structure model than from classical IAM refinement. It allows, for example, for better location of hydrogen atoms (Jha *et al.*, 2020 ▸, 2023 ▸) or detection and refinement of subtle disorder (Jha *et al.*, 2023 ▸).

Instead of using a databank, TAAM can be parametrized with multipolar parameters refined against theoretical structure factors computed directly for the studied molecule. Such parameters are not averaged between different atoms, are transferability-error free, thus they should represent a given molecule more accurately. For the purpose of this work we will call this model molecule-TAAM, also known as ‘tailored TAAM’ (Bojarowski *et al.*, 2017 ▸). To include intermolecular interactions, one can use multipolar parameters refined against theoretical structure factors computed directly for the entire crystal under study. Here we will call such a model crystal-TAAM.

Analogous reasoning can be made for the wavefunction-based methods. X-ray wavefunction refinement (XWR) allows for refinement of structural parameters and to obtain the wavefunction restrained to experimental diffraction data (Jayatilaka, 1998 ▸; Jayatilaka & Grimwood, 2001 ▸; Grabowsky *et al.*, 2012 ▸). XWR corresponds to multipole model refinement, as in both cases information about atomic and electronic structure of the crystal is extracted from the experimental data. If diffraction data are not good enough for XWR, the Hirshfeld atom refinement (HAR) can be applied. In the simple HAR approach, atomic structure parameters are refined with aspherical atomic scattering factors computed before the refinement from a gas-phase molecular wavefunction (Jayatilaka & Dittrich, 2008 ▸; Chodkiewicz *et al.*, 2020 ▸; Kleemiss *et al.*, 2021 ▸). This would correspond to molecule-TAAM refinement as, in both cases, the electronic structure of the crystal studied is not extracted from experimental data but is modelled through aspherical atom scattering factors derived from a gas-phase wavefunction computed for the single molecule forming the studied crystal. In most cases of HAR at present, the wavefunction is calculated for the molecule surrounded by the cluster of point charges and dipoles (Woińska *et al.*, 2016 ▸), or the cluster of neighbouring molecules, or a periodic boundary wavefunction is used (Wall, 2016 ▸; Ruth *et al.*, 2022 ▸). The latter may correspond to crystal-TAAM because both approaches use a crystal wavefunction, which takes into account polarization of molecular electron density due to neighbouring molecules, to precompute aspherical atom scattering factors. The major difference between any HAR and TAAM lies in the way atomic electron densities are derived from a molecular or crystal wavefunction. In HAR, a molecular or crystal electron density grid is partitioned into atomic grids using the Hirshfeld partitioning scheme; in TAAM, analytical representation of atomic electron densities are obtained through multipole model fitting in reciprocal space.

Charge densities can also be studied by the maximum entropy method (MEM) (Sakata & Sato, 1990 ▸; Nishibori *et al.*, 2007 ▸; Choudhury *et al.*, 2011 ▸), a means of obtaining a model-free electron density (or other scattering density) distribution from experimental data. The downside of the method is that it provides thermally smeared electron density, making it more difficult to compare with other techniques and theoretical simulations. Moreover, in its standard implementation it relies on the positivity of the scattering density, and although an MEM variant handling both positive and negative scattering densities exists (Sakata *et al.*, 1993 ▸), its accuracy and reliability in modelling electrostatic potential has not yet been investigated.

For 3D ED, the majority of the structures published to date are based on IAM. Multipolar scattering factors calculated from models applicable for XRD can also be applied to ED. The Mott–Bethe formula can be used to obtain electron atomic scattering factors from X-ray atomic scattering factors. We have already shown (Gruza *et al.*, 2020 ▸; Jha *et al.*, 2021 ▸) that TAAM can be applied for the refinements against 3D ED data. For theoretical data, we showed trends regarding fitting statistics and correctness of obtained geometry. However, until now, for experimental data we were not able to provide unambiguous conclusions regarding the benefits of using TAAM instead of IAM as the kinematical approach was used.

To access accurate electrostatic potential from the microcrystalline 3D ED experiments, the model of atomic scattering factors has to be combined with proper calculations of the reflection intensities, including the effects of multiple scattering. The dynamical approach significantly improves refinement results (Palatinus, Petříček & Corrêa, 2015 ▸; Palatinus, Correa *et al.*, 2015 ▸). Dynamical IAM refinement on 3D ED data allowed for detection and refinement of disordered hydrogen atoms (Palatinus *et al.*, 2017 ▸) or determination of the absolute configuration of chiral structures (Brázda *et al.*, 2019 ▸).

In this work, we present dynamical refinement of IAM with visible sub-atomic features of electrostatic potential for experimental 3D ED data with *d*_min_ = 0.56 Å. For the first time, we present dynamical TAAM refinement on experimental 3D ED data, using different sources of TAAM parametrization, and we make conclusions supported by theoretical simulations.

## 2. Methods

### 2.1. Materials

The material selected for this work is 1-methyuracil, obtained from Sigma–Aldrich and recrystallized from water for the purposes of this study. High-quality structure determination from a neutron diffraction study performed at 15, 60 and 123 K (McMullan & Craven, 1989 ▸) served as the main reference in the presented work. For comparison of the ADP values with the experimental results, the values were interpolated to 100 K.

**Figure 1:**
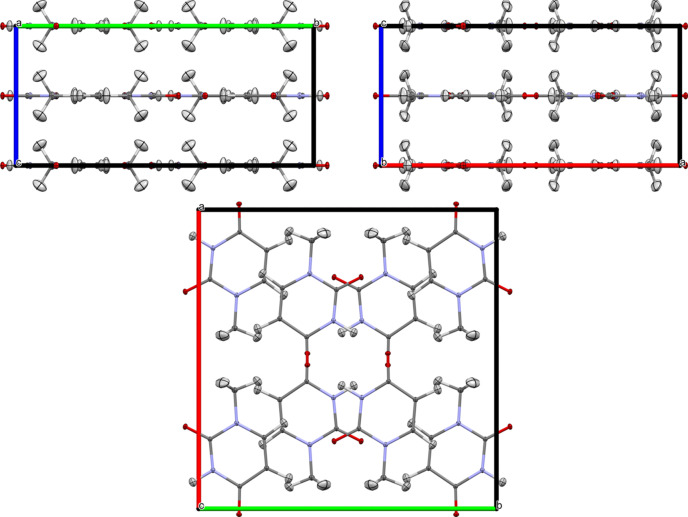
Unit-cell projections along the *a*, *b* and *c* axes; visualized in *Mercury* (Macrae *et al.*, 2006 ▸) using the 60 K reference structure from neutron diffraction.

1-methyl­uracil crystallizes in the space group *Ibam*. The molecules are arranged in layers perpendicular to the *c* axis (Fig. 1 ▸).

**Figure 2:**
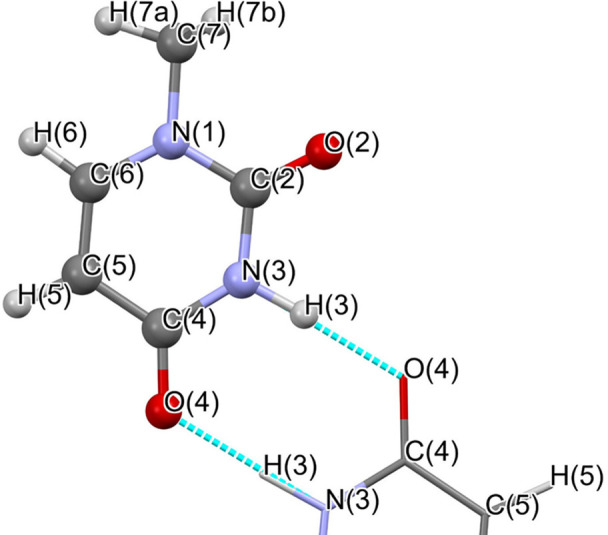
1-methyl­uracil molecule with atom names used throughout this work.

All atoms of the molecule except the hydrogen atom H7b from the methyl group are located on a special position (*x*, *y*, 0). N3—H3⋯O4 form a hydrogen bond (Fig. 2 ▸).

### 2.2. Data collection and processing

**Figure 3:**
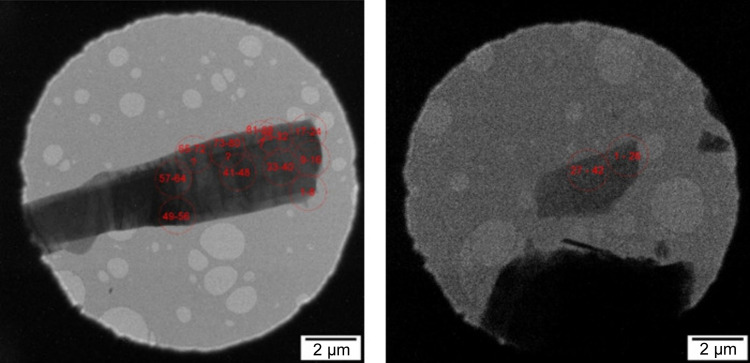
Positions of the beam on crystal 1 (left) and crystal 2 (right) during data collection.

The data were recorded about a week after the recrystallization. The crushed crystal powder was directly deposited onto the Cu holey-carbon TEM grid. Precession ED experiments were performed on an FEI Tecnai G^2^ 20 microscope with an LaB_6_ cathode equipped with an Olympus SIS Veleta CCD camera (14 bits). The accelerating voltage was 200 kV (λ = 0.0251 Å). The precession angle was 0.7°. This angle is a compromise between high data resolution on the one hand (the larger the precession angle, the less time a high-resolution reflection spends in the Bragg condition), and a complete integration of the low-resolution reflections on the other. The tilting step was 1.00°. The temperature of the tip of the sample holder was set to 100 K. Data were processed using *PETS2* (Palatinus *et al.*, 2019 ▸). Data from many crystals were collected. The material was electron beam sensitive, so it was necessary to spread the dose into a larger volume by shifting the beam over the crystal (Fig. 3 ▸). The electron doses for crystals 1 and 2 were 0.28 and 0.05 e Å^−2^ per frame, respectively. It was possible to measure 8 and 26 frames at a particular point of the crystal, respectively, to limit the influence of beam damage. This influence was followed by a decrease in the frame-by-frame apparent magnification correction (Brázda *et al.*, 2022 ▸). The beam damage was considered acceptable when the magnification correction was less than 0.1% with respect to the first frame measured on a given point of a crystal. Out of these, one dataset (No. 1) stood out as the best and was selected for further analysis. However, the data completeness was too low, and the dataset needed to be augmented with data from a second crystal (No. 2) (Fig. 3 ▸). Altogether, 103 frames from several spots on the crystals were included in the refinement.

The dynamical refinement was performed using *Jana2020* (Petříček *et al.*, 2023 ▸). Analysis of the refinement showed significant discrepancies in the fit of several frames of the second crystal, especially those close to the [001] axis. The reason for a compromised fit of these frames lies in the crystal imperfections. Calculation of the dynamical effects assumes a perfect crystal, yet when we look at the crystals in Fig. 3 ▸ we can see bend contours indicating a mosaic crystal. Since the dynamic effects are greatest in the zone axes, frames measured in and near this crystal orientation have the worst *R* factors. To avoid biasing the result by the poor fit of these frames, 15 frames were omitted from the data of the second crystal, which had the worst *R* factors. These high *R* factors indicate that crystal 2 is probably a less ideal crystal than crystal 1. All frames of the first crystal including the zone-axis ones were used. The final completeness of the data up to *d*_min_ = 0.56 Å was 63%. This is still a low completeness, but as the molecule is oriented in the *xy* plane, and this plane is in the centre of the coverage range, the data provide sufficient coverage to extract the features in the plane of the molecule.

In the following chapters, these data are referred to as experimental structure factors, 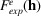 , and are the core of the presented work.

### 2.3. Theoretical structure factors

Theoretical structure factors were derived from DFT calculations of the molecular or crystal structure. The purpose of the theoretical structure factors was twofold. First, they were used to generate the parameters for various TAAM parametrizations, *i.e.* to obtain models of electronic structures. Second, they were used to validate the actual crystal atomic structure refinement and compare it with the refinement against experimental data.

(1) X-ray structure factors corresponding to a static isolated 1-methyl­uracil molecule structure are denoted 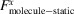 . For the calculation of 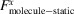 , the molecule with the geometry taken from the neutron diffraction experiment at 15 K (McMullan & Craven, 1989 ▸) with ADPs set to 0 was placed in the centre of a *P*1 unit cell, *a* = *b* = *c* = 30 Å, α = β = γ = 90°. DFT calculation of the electron density was performed at the B3LYP/6-31G** level using *GAUSSIAN16* (Frisch *et al.* 2016 ▸), with fixed molecular geometry, the *X*—H bond lengths shifted to average values from neutron diffraction (Allen & Bruno, 2010 ▸). Only valence electrons were included in the 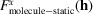 calculations. Structure factors up to the resolution *d*_min_ = 0.45 Å were generated from the computed electron density. These settings correspond to the settings used for creating the UBDB (Kumar *et al.*, 2019 ▸). These structure factors were used to generate the multipole parameters for molecular valence electrons, and after combination with the core electron term of the multipole model (frozen-core approximation), it generated molecule-TAAM.

(2) X-ray structure factors corresponding to the static 1-methyl­uracil crystal structure are denoted 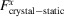 . To obtain 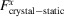 , the reference 15 K crystal structure of 1-methyl­uracil (McMullan & Craven, 1989 ▸) was used with ADPs set to 0. Single-point calculations were performed by applying periodic DFT calculations implemented in *CRYSTAL17* (Dovesi *et al.*, 2017 ▸). The B3LYP functional augmented with an empirical dispersion term as proposed by Grimme (2006 ▸) and modified for molecular crystals (Civalleri *et al.*, 2008 ▸) with POB-TZVP basis set (as implemented in *Crystal17*) were used. The level of accuracy in evaluating the Coulomb and exchange series was controlled by five TOLINTEG parameters for which values of 10^6^, 10^6^, 10^6^, 10^7^ and 10^29^ were used. The shrinking factors (IS) along the reciprocal-lattice vectors were set to 4. The level shifter value was set to 0.6 hartree. The condition for the self-consistent field (SCF) convergence was set to 10^−7^ on the total energy difference between two subsequent cycles. Data up to the resolution *d*_min_ = 0.37 Å were generated by *CRYSTAL17*. The structure factors generated from this electron density were used to obtain parameters for crystal-TAAM.

(3) 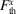 were calculated in the same way as 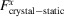 , but with ADPs from the reference 15 K crystal structure included in the calculation using dedicated *CRYSTAL17* functionality (Erba *et al.*, 2013 ▸). These structure factors represent theoretical, perfect, noise-free X-ray diffraction data from the crystal structure including ADPs. In this work, they were used only to generate theoretical ED structure factors.

(4) 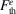 are theoretical structure factors for ED. They were calculated from 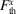 by applying the Mott–Bethe formula using the *DiSCaMB* software. Data up to the resolution *d*_min_ = 0.6 Å were used to test various refinement models against perfect, noise-free kinematical ED data.

### 2.4. HC-MM refinements against theoretical *F^x^*_molecule–static_ and *F^x^*_crystal–static_ structure factors

Hansen–Coppens multipole model (HC-MM) (Hansen & Coppens, 1978 ▸) refinements were performed to obtain parameters for molecule-TAAM (from 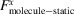 ) and crystal-TAAM ( 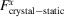 ). The refinements were performed using *XD2006* (Volkov *et al.*, 2006 ▸). Multipole model parameters, populations and contraction–expansion parameters (*P*_val_, *P_lm_*, κ, κ′), were refined up to hexadecapoles for non-hydrogen atoms and up to quadrupoles for hydrogen atoms. Atomic coordinates were fixed to the target values (*i.e.* the values used to compute theoretical structure factors), ADPs were fixed to 0 and the scale was fixed to 1. The symmetry of all non-hydrogen atoms was set to ‘m’ except for the methyl carbon, which was set to ‘3m’ and only symmetry-allowed multipolar parameters were refined. All hydrogen atoms had assigned cylindrical symmetry (only bond-directed multipoles). Refinement was performed against |*F*(**h**)| (here equal to 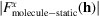 or 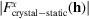 , respectively) with constrained phases, with unit weights, against all reflections. For 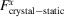 , parameters from the UBDB were used as a starting point. The refinement strategy was exactly the same as for creating UBDB. In the refinement against 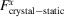 , the κ′ parameters of the nitro­gen atoms did not converge to realistic values and were therefore constrained to values from the UBDB.

Multipolar parameters and local coordinate systems are provided in Tables S1, S2 and S3 of the supporting information.

### 2.5. Refinements against theoretical *F^e^*_th_ structure factors

*Olex2* 1.3 (Dolomanov *et al.*, 2009 ▸) coupled with the *DiSCaMB* library (Jha *et al.*, 2020 ▸) was used for the refinements. The electron scattering factors from Table 4.3.2.3 of *International Tables for Crystallography* Vol. C (Cowley *et al.*, 2006 ▸) were used for the IAM refinements. The UBDB2018 databank (Volkov *et al.*, 2004 ▸; Dominiak *et al.*, 2007 ▸; Jarzembska & Dominiak, 2012 ▸; Kumar *et al.*, 2019 ▸), together with LSDB (Volkov *et al.*, 2004 ▸), was used as a source of parameters for TAAM. For molecule-TAAM and crystal-TAAM refinements, multipolar parameters were taken from the HC-MM refinements against 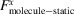 and 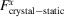 , respectively. The *DiSCaMB* library was used for computing electron form factors through the Mott–Bethe formula applied to the X-ray form factors calculated directly from multipole models. All refinements were performed against |*F*(**h**)|^2^ (here equal to 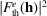 ). The constant value of σ(*F* ^2^) = 1.0 was assigned to |*F*(**h**)|^2^. The following weighting scheme was used: 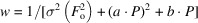 , where *P* = 1/3 × maximum of (0 or 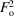 ) + 2/3 × 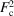 , 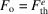 and 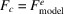 , which was computed from the refined model. Optimal values for the *a* and *b* parameters were automatically calculated in order to achieve a normal distribution of residuals. All atomic coordinates and ADPs were freely refined. Non-hydrogen atom ADPs were treated anisotropically. Two refinements were performed, one with isotropic hydrogen atom ADPs and one with anisotropic hydrogen atom ADPs. As the theoretical structure factors were obtained by a Fourier transform of the electron density, the kinematical refinement approach is appropriate and was used. Only *R*_all_(*F*) was considered in further analyses, as *R*_obs_(*F*) for refinements with a constant value of σ(*F* ^2^) are meaningless. To facilitate a detailed comparison with the experimental results, the following sections present the results of theoretical simulations for a unit cell selected in a manner analogous to that performed for the experimental data presented in this work. This means that the original unit cell of the 15 K 1-methyl­uracil reference crystal structure (McMullan & Craven, 1989 ▸) has been transformed by the [0 1 0 1 0 0 0 0 −1] matrix (the *a* and *b* directions have been swapped).

### 2.6. Dynamical refinement against experimental *F^e^*_exp_ structure factors

Experimental data are affected by the dynamical diffraction effects, and therefore the refinement must take these effects into account to produce the best result. For the dynamical refinement, *Jana2020* (versions 1.3.30 and 1.3.36) was used. The same multipole model parameters for TAAM, molecule-TAAM and crystal-TAAM were used as in the refinement against theoretical data. The Mott–Bethe formula was used to obtain electron form factors from the X-ray form factors calculated directly from TAAM. All dynamical refinements were performed against |*F*(**h**)| (here equal to 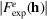 ). All reflections with a resolution up to *d*_min_ = 0.56 Å, maximal relative excitation error *RS*_g_ = 0.75 (Palatinus, Correa *et al.*, 2015 ▸) and with maximal excitation error *S*_g_ = 0.02 Å^−1^ were included in the refinement. All atomic coordinates including hydrogen atoms were refined freely. The non-hydrogen atom displacement parameters were refined anisotropically, and all hydrogen atoms ADPs were refined isotropically. The crystal thickness was refined for each crystal separately. *R*_obs_(*F*) was calculated for reflections with *I* > 3σ(*I*).

## 3. Results and discussion

### 3.1. Comparison of various models with the theoretical structure factors and electrostatic potential

As a basic reference, we compared the electrostatic potentials and the structure factors of individual models (IAM, TAAM, molecule-TAAM, crystal-TAAM) with the ideal theoretical electrostatic potential and the corresponding structure factors 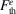 . No structure refinement is involved in this step. All 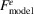 were computed by applying a particular scattering model (IAM, TAAM, molecule-TAAM, crystal-TAAM) to the target values of atom coordinates and ADPs, *i.e.* the same values that were used to compute 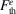 .

#### 3.1.1. Structure factor amplitudes

**Figure 4:**
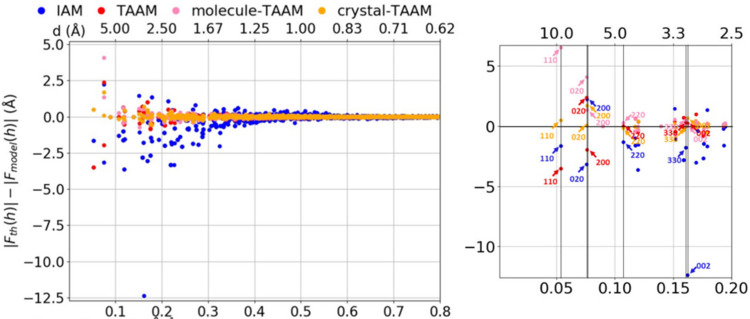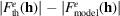 (Å) versus resolution [1/2*d* (Å^−1^), *d* (Å)] for IAM, TAAM, molecule-TAAM and crystal-TAAM. All 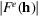 were computed using the target crystal structure (atomic positions and thermal parameters used to compute 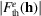 ).

The plot of 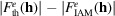 as a function of resolution (Fig. 4 ▸) reveals systematic discrepancies between IAM and the results of periodic DFT calculations. At low resolution, the differences are mostly negative, whereas they tend to be smaller and mostly positive at intermediate resolutions. The most problematic for IAM are the 002, 130, 020, 231 and 321 reflections from the absolute scale point of view (Fig. 4 ▸, Table S4 in the supporting information) and 200, 130, 310 and 321 from the relative scale (Table S5). For all TAAM versions, the differences from the periodic DFT results become much smaller for the majority of the structure factors. The improvement is the most prominent at low resolution, but also medium resolution structure factors are improved. None of the TAAM versions have problems with the 002 reflection. Nevertheless, at low resolutions, up to *d* ≲ 3 Å, some large 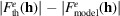 differences are still observed for TAAM and molecule-TAAM. The biggest absolute discrepancies are for the 110 and 200 reflections with too high 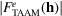 amplitudes and for the 020 reflection with a too low 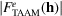 amplitude (Fig. 4 ▸, Table S5). Among them, the 110 reflection computed with TAAM disagrees with the target value (*i.e.* the value of 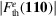 ) more than in the case of IAM. When using multipolar parameters calculated specifically for the 1-methyl­uracil molecule in the gas phase (molecule-TAAM), the 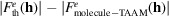 residues have mostly positive values, and some of them are among the largest observed for all the tested scattering models. The biggest discrepancies in absolute values are for reflections 110 and 020, and in addition for 200 reflection when a relative scale is included (Fig. 4 ▸, Table S5). The 110 reflection computed with molecule-TAAM disagree with the target value even more than for TAAM. In fact, molecule-TAAM is the worst among all methods in modelling the 110 reflection. The effect of changing the sign of the residues for TAAM and molecule-TAAM is also visible in the Fourier residual potential maps (Fig. 6). Inclusion of the crystal environment during the preparation of the multipole model parameters (crystal-TAAM) removes the problem of 110 and 020 reflections – the structure factors amplitudes from crystal-TAAM give nearly perfect agreement. There is only one structure factor from crystal-TAAM where the amplitude has a visibly wrong value, it is the one for the 200 reflection.

To surmise, for resolutions higher than 5 Å, all TAAM versions give similarly good 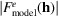 values. The significant variability for the very low-resolution reflections is likely due to their high sensitivity to even small differences in the electrostatic potential distribution. This sensitivity is likely to be responsible for the differences in the crystal atomic structure refinements performed with the use of various TAAMs (see also below).

Numerical values for all marked reflections and a plot with 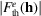 versus resolution are provided in Table S4 and Fig. S1 of the supporting information. It can be compared with the analogical figure given by Gruza *et al.* (2020 ▸).

#### 3.1.2. R(*F*)

For the target atomic model *R*_all_(*F*) values are equal to 3.28, 0.91, 0.88 and 0.71% for IAM, TAAM, molecule-TAAM and crystal-TAAM, respectively. More than 2 percentage points improvement is observed when changing the scattering model from IAM to any TAAM version. One order of magnitude smaller differences are observed in global *R*_all_(*F*) values computed for various TAAM.

#### 3.1.3. *R*(*F*) versus resolution

**Figure 5:**
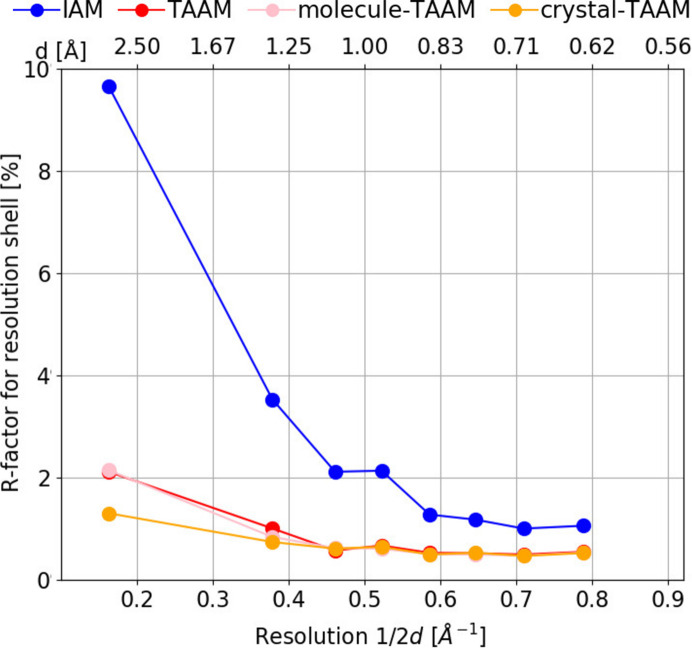
Mean *R*_all_(*F*) (%) for eight resolution shells calculated for various scattering models (IAM, TAAM, molecule-TAAM, crystal-TAAM) applied to the target crystal structure. 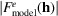 were computed using the target atomic positions and thermal parameters (*i.e.* the same values which were used to compute 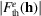 ).

The difference between IAM and TAAM is observed for all resolution shells (Fig. 5 ▸). For the lowest-resolution shell, the difference is 7.5 percentage points, for other shells between 2.5 and 0.5 percentage points. There are no noteworthy differences between TAAM and molecule-TAAM (the biggest difference is 0.2 percentage points). Differences between TAAM and crystal-TAAM are visible only for the first two shells and are less than 1 percentage point.

#### 3.1.4. Residual electrostatic potential

**Figure 6:**
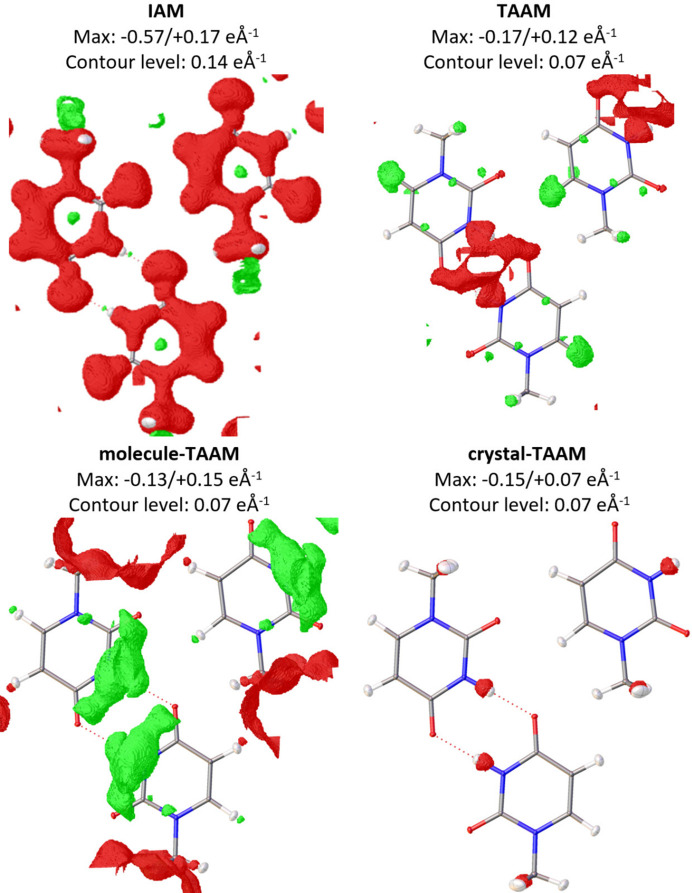
Residual potential maps 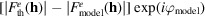 for IAM, TAAM, molecule-TAAM and crystal-TAAM applied to the target crystal structure. Contour colour code: green – positive, red – negative. Thermal ellipsoids with 50% probability. Note that for IAM the contour level is twice the size of the other models.

Fig. 6 ▸ shows the Fourier difference potential maps for each of the four tested models. For IAM, the negative residual potential located at the atoms and bonds dominates the map. This negative potential disappears upon the change from IAM to TAAM. The largest negative residual appears now only around the region of hydrogen bonds. This is expected, as intermolecular interactions are not modelled by TAAM. For molecule-TAAM negative residues are further lowered, but the positive ones are larger than for TAAM. Positions of residuals are similar to TAAM, except broad negative residues on the hydrogen bond area now are positive. Apparently, the electrostatic potential of the 1-methyl­uracil molecule modelled with a tailored approach differs slightly from the one approximated with the databank approach, especially in the hydrogen atom position regions. Crystal-TAAM results in a much cleaner residual potential map, indicating that the multipole parameters of that model describe reasonably well also the intermolecular hydrogen bonding interactions. However, the description is not perfect, and a small negative potential remains on the atom H3 involved in the hydrogen bond.

Interestingly, removing just the three worst-matching reflections from the calculation of the difference potential maps (110, 200, 020), the maps for TAAM and molecule-TAAM clean significantly. These reflections are thus the most strongly affected by the tiny differences in the electron density deformations (and thus electrostatic potential deformations) due to intermolecular interactions. Maps obtained without these three reflections (*i.e.* maps obtained with the corresponding three Fourier coefficients set to zero) are presented in Fig S2.

### 3.2. Crystal atomic structure refinement against simulated data

Refinements of atomic structure parameters (coordinates, atomic displacement parameters) against simulated data (theoretical structure factors 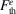 ) improved the fit of the model to the data as indicated by the residual potential maps and, in some cases, by *R*_all_(*F*), but the parameters of the atomic structure departed from the target values (*i.e.* values used to compute 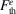 ). It is interesting to observe the magnitude of the changes, depending on the scattering model applied. In all the refinements presented here, all atoms were refined freely with anisotropic atomic displacement parameters.

#### 3.2.1. The worst-matching reflections

Similarly, as was observed for the target atomic model, among the worst-fitted low-angle reflections for the IAM refinement there are mostly too strongly modelled reflections: 220, 310, 130, 400, 040, 321, 231, 330 and 002 (Table S6). A visible overall improvement is observed for all TAAM refinements. 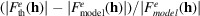 values are closer to zero after the refinement than they were for the target atomic model (*i.e.* yet unrefined model), but all other trends remain the same.

#### 3.2.2. *R*_all_(*F*)

*R*_all_(*F*) is equal to 3.24, 1.1, 1.1 and 0.83% for the IAM, TAAM, molecule-TAAM and crystal-TAAM refinements, respectively. Only when IAM was used, the refinement led to slightly lower *R*_all_(*F*) values compared with the target atomic model. In none of the TAAM cases, the *R*_all_(*F*) after refinement was smaller than that for target structure, though crystal-TAAM allowed us to achieve slightly better fit than the other two TAAM versions. Nevertheless, the differences between *R*_all_(*F*) values from refined and unrefined parameters of atomic structures are very small.

#### 3.2.3. *R*(*F*) versus resolution

**Figure 7:**
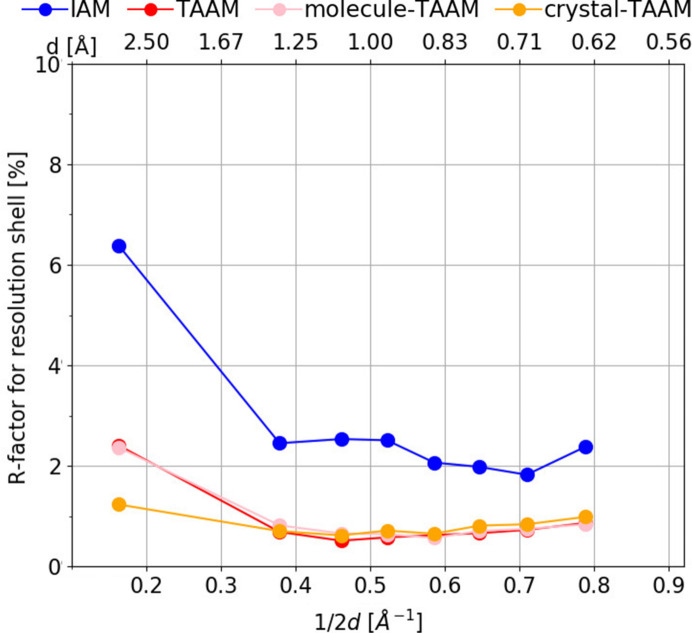
Mean *R*_all_(*F*) (%) for eight resolution shells calculated from kinematical refinements on simulated 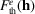 kinematical data. Anisotropic ADPs were applied to all atoms.

Similarly to the comparison without refinement, after refinement there is a difference between IAM and TAAM observed for all resolution shells (Fig. 7 ▸). There are no differences worth noting between molecule-TAAM and TAAM. Differences between crystal-TAAM and TAAM are visible only for the lowest-resolution shell.

#### 3.2.4. Residual electrostatic potential

**Figure 8:**
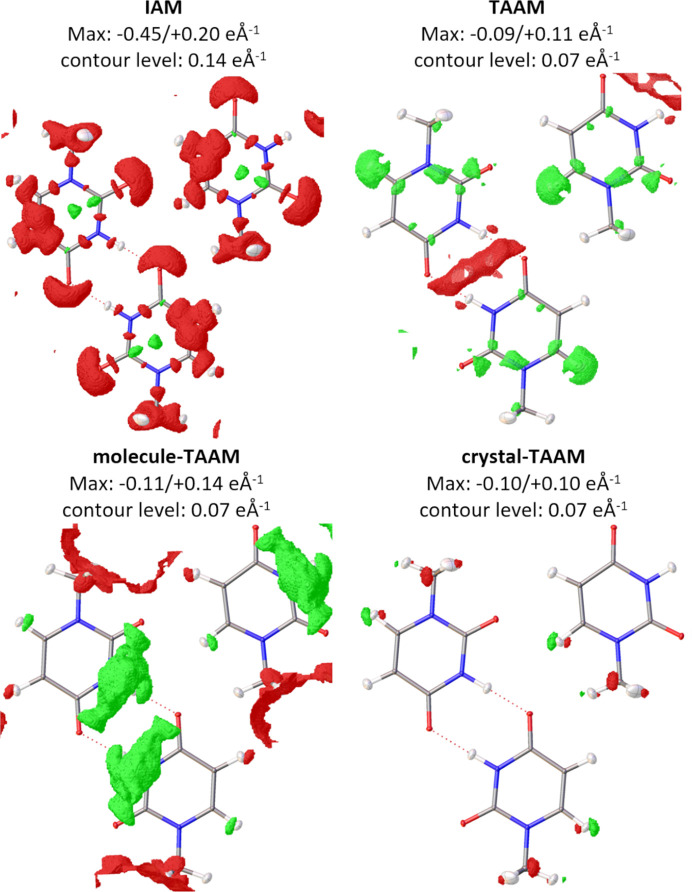
Residual potential maps 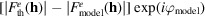 from kinematical refinements on simulated 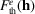 kinematical data with various scattering models: IAM, TAAM, molecule-TAAM and crystal-TAAM. Contour colour code: green – positive, red – negative. Anisotropic ADPs were applied to all atoms. Thermal ellipsoids with 50% probability. Note that for IAM the contour level is twice the size of the other models.

For the IAM refinement, the minimum and maximum residual potential peaks are −0.45/+0.20 e Å^−1^ (Fig. 8 ▸). The negative residues at atom positions are visibly smaller than they were before the refinement, but large negative residues on covalent bonds and at the lone electron pair regions remained. For TAAM, the minimum and maximum residues drop to −0.09/+0.11 e Å^−1^. Negative residual potential on covalent bonds disappeared in the TAAM refinement (compared with the IAM refinement), or became slightly positive. The positive peak at the H6 atom and broad negative residues in the hydrogen-bond area involving the H3 atoms are still very visible compared with before the refinement. For molecule-TAAM refinement, there are still visible broad residues in the hydrogen-bond area, they are still positive as opposed to TAAM. The maximum residues are −0.11/+0.14 e Å^−1^. For crystal-TAAM, the maximum residues remain very similar at −0.10/+0.10 e Å^−1^. Most importantly, crystal-TAAM maps are as clear as the maps calculated for the target atomic structure (Fig. 6 ▸). Thus, any scale effect on the appearance of the residual maps from various TAAM can be excluded. The improvement of the residual potential map on introducing crystal-TAAM, which remains the highest after crystal atomic structure refinement, again highlights the importance of considering the specific intermolecular interactions in the structure to obtain the best fit.

With experimental data it was not possible to apply anisotropic ADPs to hydrogen atoms and isotopic ADPs were used, see Section 3.3. For the direct reference to the refinements on experimental data, we also performed refinements on simulated data with the isotropic model of atomic displacement parameters for hydrogen atoms, for more details see Figs. S3, S4 and S5.

#### 3.2.5. *X*—H bond lengths

**Figure 9:**
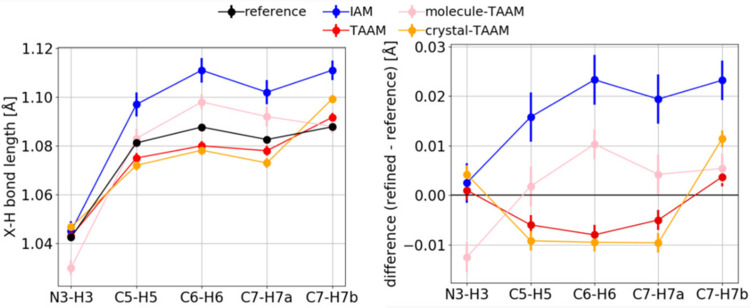
Left: *X*—H bond lengths (Å), error bar equal to one e.s.d. Right: *X*—H_refinement_ − *X*—H_reference_, error bar calculated from error propagation. All data from kinematical refinements against simulated 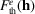 kinematical data. Anisotropic ADPs were applied to all atoms. Reference: target values from neutron data at 15 K.

*X*—H bond lengths from TAAM refinement are closer to the reference target values (*i.e.* values calculated from coordinates used to compute simulated 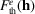 structure factors) than those from IAM, and the difference is visible in each *X*—H length and in the mean error (ME) and root-mean-square deviation (RMSD) values (Table 1 ▸, Fig. 9 ▸). The ME values are 0.018 and −0.003 Å for IAM and TAAM, respectively. This means that lengths from IAM are systematically too long, while those from TAAM are slightly too short, but the discrepancies from TAAM are of the same order of magnitude as uncertainties on the bond lengths. The *X*—H bond lengths from molecule-TAAM refinement are slightly too long on average, but the ME is of the same order of magnitude as the uncertainties and the RMSD is comparable to other TAAM versions. Crystal-TAAM refinement results in bond lengths comparable to TAAM.

#### 3.2.6. ADPs

**Figure 10:**
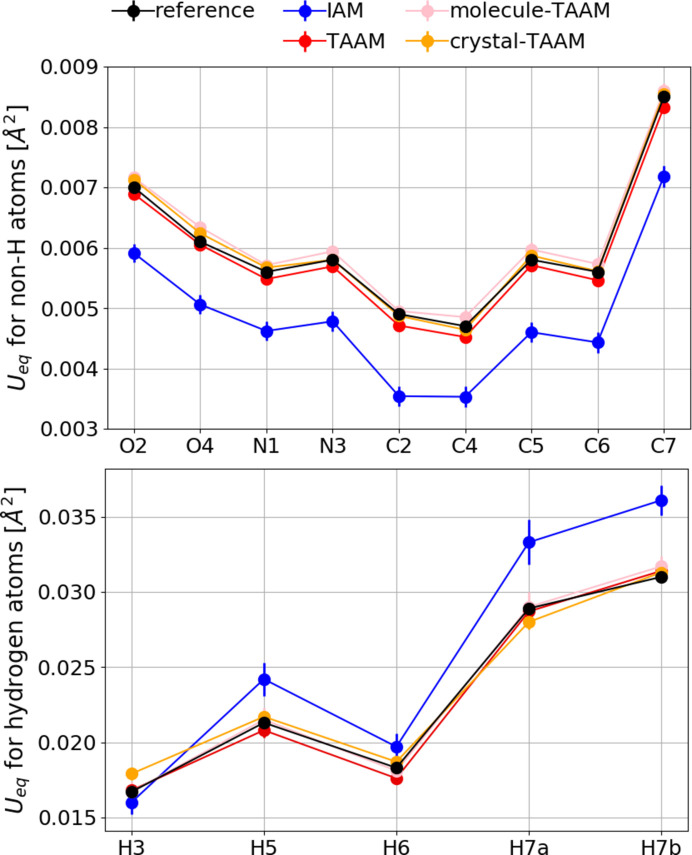
*U*_eq_ for non-hydrogen atoms (top) and *U*_eq_ for hydrogen atoms (bottom) (Å^2^) with uncertainties. All data are from kinematical refinements against simulated 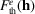 kinematical data. Anisotropic ADPs were applied to all atoms. Reference: values from neutron data at 15 K.

The ADPs for non-hydrogen atoms are systematically too small for IAM with an ME of *U*_eq_ = −0.00115 Å^2^ (Fig. 10 ▸, Table S7), meaning they are *ca.* 20% underestimated. The error improves by an order of magnitude to −0.00013 Å^2^ for TAAM. Values of *U*_eq_ for non-hydrogen atoms are very well refined with all TAAM versions with differences |*U*_refinement_ − *U*_reference_| comparable to the e.s.d., with RMSDs of 0.00014 Å^2^, 0.00015 Å^2^ and 0.00008 Å^2^ for TAAM, molecule-TAAM and crystal-TAAM, respectively. A similar result is obtained for the ADPs of the hydrogen atoms with an ME of *U*_eq_ = 0.0026, −0.0002, 0.0002 and 0.0003 Å^2^ for IAM, TAAM, molecule-TAAM and crystal-TAAM, respectively; however, this time the *U*_eq_ values from IAM are systematically too large, by *ca* 10%. Too-small ADPs of non-hydrogen atoms from the IAM refinement probably result from the compensation effect. The excess of electrons at the lone electron pair and bonding regions of a molecule generate an extra negative aspherical electrostatic potential in the valence region of atoms, which is not modelled by IAM. The transfer of electrons from the atom to the bonding region also increases the positive potential close to the nucleus. To compensate for less positive electrostatic potential in the bonding regions and more positive potential at the nucleus, the ADPs become smaller in IAM. The behaviour of ADPs for hydrogen atoms is more complicated and is hard to explain with one single effect. Electrostatic potential for free neutral hydrogen atoms resulting from IAM is either too little positive (too many electrons) or too contracted (too much expanded electron density), and it must be artificially diffused by too-large ADPs.

### 3.3. Dynamical refinement against experimental data

With experimental data we performed both dynamical and psuedokinematical refinements. With pseudokinematical refinement we were unable to achieve acceptable quality of crystal atomic structure without applying heavy restraints and constrains to atom positions and ADPs. Refinement of hydrogen atom positions was unstable and many ADPs for non-hydrogen atoms became non-positive definites (Fig. S6). The overall *R*_obs_(*F*) and *R*_all_(*F*) values for IAM pseudokinematical refinement were very high, 20.60 and 26.58%, respectively. A dynamical approach substantially improved the results of the refinement. The detailed analysis of dynamical refinements with the application of IAM, TAAM and crytal-TAAM scattering models is given below. Molecule-TAAM was not used as no improvements over TAAM are expected based on theoretical simulations.

#### Comparison of 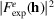 and 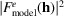


3.3.1.

**Figure 11:**
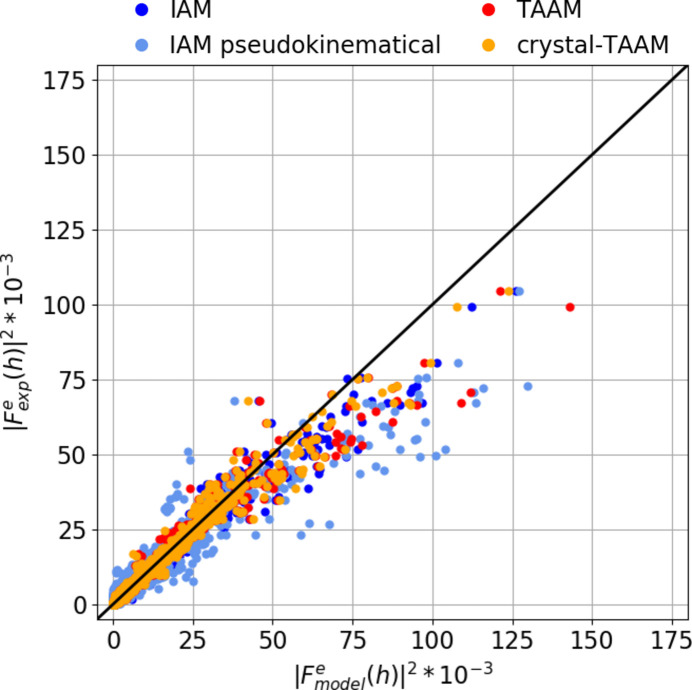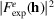 versus 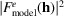 from dynamical refinements on experimental data with various scattering models: IAM (blue), TAAM (red) and crystal-TAAM (orange). The black line shows values of 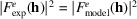 .

Fig. 11 ▸ shows the plot of 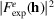 against 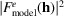 . One can see a good match especially at low intensities. A significant discrepancy towards too low 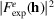 (or too high 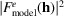 in other words) is visible for many higher intensity reflections. The discrepancy is the largest for IAM and improves visibly on the introduction of TAAM or crystal-TAAM modelling, but does not vanish completely. It is likely that some unmodelled effects of dynamical scattering still remain.

Comparing the worst fitted reflections in the dynamical refinements, it appears that these are mostly the lowest-angle reflections (Table 2 ▸). For IAM dynamical refinement, reflections from the {130}, {231} and {321} groups of symmetry-equivalent reflections are among the worst fitted reflections, followed by {200} and {020}. With TAAM, the {110} and {020} reflections become the worst, followed by {220} and {330}, whereas for {130}, {231}, {321} and {200} the fitting improves. For crystal-TAAM, the fit for the {220}, {200} and {020} reflections worsens a bit more compared with IAM, but the remaining reflections are much better fitted, including {110}. Compared with TAAM, the biggest improvement with crystal-TAAM is observed for the {110} and {330} reflections.

Note that reflections from the *hk*0 zone are mostly present only in the dataset for crystal 1 and that the {002} reflection is not present in any of the two datasets.

The problems with proper modelling of the {110} reflections by TAAM agree very well with the theoretical simulations. According to the latter, these are the worst matching TAAM reflections on both scales, absolute and relative (see Fig. 4 ▸, Table S5), and their intensities are overestimated by TAAM (see Fig. 4 ▸, Table S5). IAM predicts intensity of the {110} reflection slightly better, also according to theoretical simulations. The problem with the {110} reflection is solved when crystal-TAAM is applied, both in the refinement against experimental data and in the refinement against simulated data.

The observation that the {110} reflection for the IAM refinement is less problematic than the {200} and {020} reflections also agrees with theoretical simulations, according to which the latter two are the two worst-matching IAM reflections on an absolute scale among the experimentally observed.

According to theoretical simulations, the {200} reflection is problematic to model by all the three applied models IAM, TAAM and crystal-TAAM. IAM and crystal-TAAM underestimate its intensity, whereas TAAM overestimates it. These predictions agree with experimental results to the full extent for IAM and crystal-TAAM dynamical refinements, and only partially for TAAM. The first two experimental refinements show a relatively bad fit underestimating the intensity, whereas TAAM with higher {200} intensity does not overestimate it but fits the experiment.

The behaviour of the {020} reflections in experimental refinements is well predicted by the simulations for IAM but poorly for TAAM and crystal-TAAM. According to them, only IAM should overestimate it, TAAM should underestimate it and crystal-TAAM should fit well. Also the experimental results for the {220} and {330} reflections do not agree with the simulations. TAAM and crystal-TAAM should not have problems with modelling their intensities, and IAM should overestimate their intensities. Some other factors, not taken into account by the simulations, must play a role in experimental data.

The observation that the fit of {130}, {321} and {231} reflections improves after switching from IAM to TAAM or crystal-TAAM is consistent with theoretical simulations. These are reflections which are very well described by both versions of TAAM and their intensities are overestimated by IAM.

#### 3.3.2. R(*F*)

For the IAM dynamical refinement, overall *R*_obs_(*F*) is equal to 9.53% (Table 3 ▸). This can be considered quite a good fit for the refinement against 3D ED data from an organic crystal. *R*_obs_(*F*) improves with changing to TAAM, for which it is 8.92% (0.61 percentage points difference). The effect of changing the scattering model is even more visible for *R*_all_(*F*), with values of 13.55 and 12.73% for IAM and TAAM, respectively (0.82 percentage points difference). This means that the improvement is larger for weaker reflections. *R*(*F*) statistics are further lowered when switching to crystal-TAAM, *R*_obs_(*F*) = 8.75% and *R*_all_(*F*) = 12.57%. Interestingly, data from crystal 1 respond slightly differently to the changing of scattering model than data from crystal 2. For crystal 1, application of crystal-TAAM allows us to achieve a visibly better fitting than TAAM, for crystal 2 there is almost no difference in *R*(*F*) statistics between TAAM and crystal-TAAM. This can be attributed to the overall lower quality of data from crystal 2, with systematic errors masking the potential improvement of an improved model. This can also be enhanced by the fact that almost no {110} reflections are present in the dataset for crystal 2, and there are many of them in the dataset for crystal 1. The {110} reflections are badly modelled by TAAM and quite well by crystal-TAAM.

Change of *R*_all_(*F*) from IAM to TAAM refinement is equal to 0.82 percentage points for experimental dynamical refinements and 2.14 percentage points for theoretical refinements. The difference in *R*_all_(*F*) between TAAM and crystal-TAAM is equal to 0.2 percentage points for experimental and 0.2 for theoretical refinements. Although absolute values of *R*_all_(*F*) for experimental refinements are several percentage points higher than for theoretical simulations, the differences between scattering models are quite consistent.

#### 3.3.3. *R*(*F*) versus resolution

**Figure 12:**
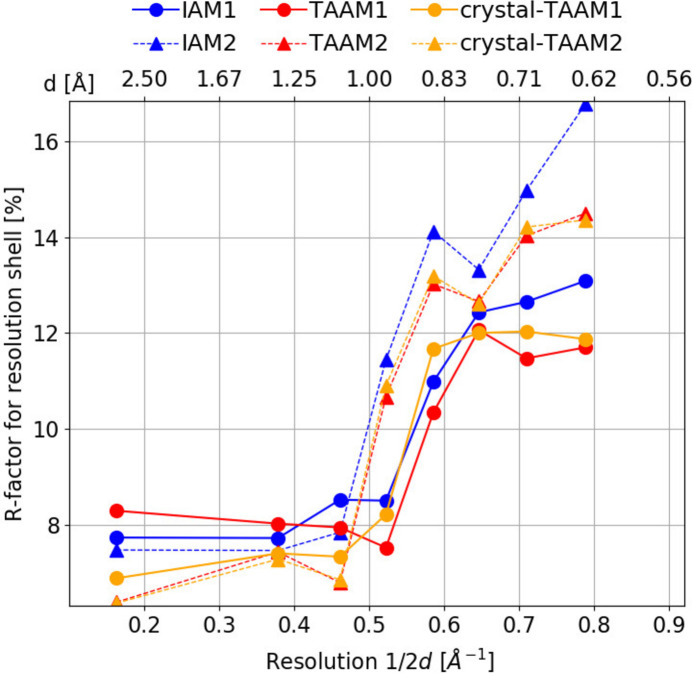
Mean *R*_obs_(*F*) (%) for eight resolution shells calculated from dynamical refinements on experimental data. Solid lines and dotted lines are the values calculated for the data from crystals 1 and 2, respectively.

As in the case of the simulated data, the low-angle reflections in the experimental data also have a different sensitivity to the applied scattering model than the high-angle reflections (Fig. 12 ▸). *R*_obs_(*F*) values for IAM dynamical refinement are, almost without exception, larger than for TAAM and crystal-TAAM, irrespective of resolution or whether data for crystals 1 or 2 are analysed. Only in the case of the two lowest-resolution shells of data from crystal 1, the highest value of *R*(*F*) is observed for TAAM. For *R*(*F*) from crystal-TAAM, no problem with fitting to low-angle reflections is visible. In further resolution shells, TAAM and crystal-TAAM have similar *R*_obs_(*F*) values, always lower than those from IAM. This indicates that the experimental data are good enough to discriminate between differences in TAAM and crystal-TAAM scattering models, in which differences appear to be present in the low-angle reflections only. Why IAM seems to perform better than TAAM in the lowest-resolution shell for crystal 1, though in all the remaining resolution shells is worse, remains unclear. Possibly, the [001] zone-axis reflections, or some of them, are very sensitive to the details of TAAM, the sensitivity being enhanced by dynamical effects. The sensitivity causes TAAM, which is not highly accurate to describe broad features in the electrostatic potential, to give a worse fit to some of the low-angle reflections than IAM. The problem is solved by crystal-TAAM. The problem is visible only for crystal 1, because the set of reflections from crystal 2 used during the refinement did not contain the zone-axis data as explained in the Methods[Sec sec2].

#### 3.3.4. Residual electrostatic potential

**Figure 13:**
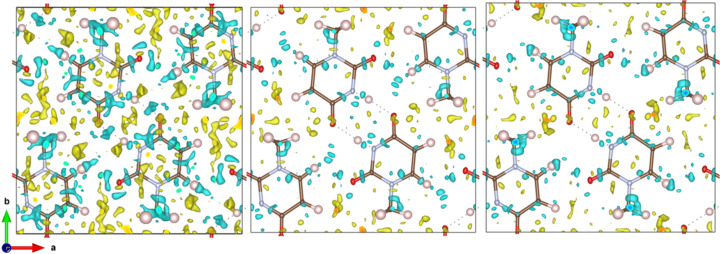
Residual potential maps 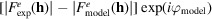 for dynamical refinements on experimental data with various scattering models: IAM (left), TAAM (middle) and crystal-TAAM (right). Contour level = 0.14 e Å^−1^; cyan: negative, yellow: positive. Thermal ellipsoids with 50% probability.

The residual potential map after IAM dynamical refinement on experimental data appears quite noisy (Figs. 13 ▸ and S7). The maximum negative and positive potential residues are −0.39/+0.31 e Å^−1^. However, on closer inspection, it has features remarkably similar to the residual potential obtained against simulated theoretical data (Figs. 6 ▸ and 8 ▸), namely negative residues located in the bonding areas and in the expected lone-pair regions of the oxygen atoms (Fig. 13 ▸). Also, the largest positive potential is located in both cases on the C2 atom.

A much cleaner potential map is obtained with TAAM dynamical refinement, with maximum negative and positive residues −0.29/+0.24 e Å^−1^. Most of the residues visible for IAM are lowered with TAAM, especially positive residues around C2 and negative residues on covalent bonds and around the O2. Crystal-TAAM dynamical refinement further lowered the maximum residual potential extremes to −0.29/+0.22 e Å^−1^, but in comparison with TAAM, the difference is very small. Opposite to the results shown for simulated data, it is hard to observe any systematic difference between residual maps from TAAM and crystal-TAAM dynamical refinements on experimental data. The tiny details are possibly hidden in the noise.

**Figure 14:**
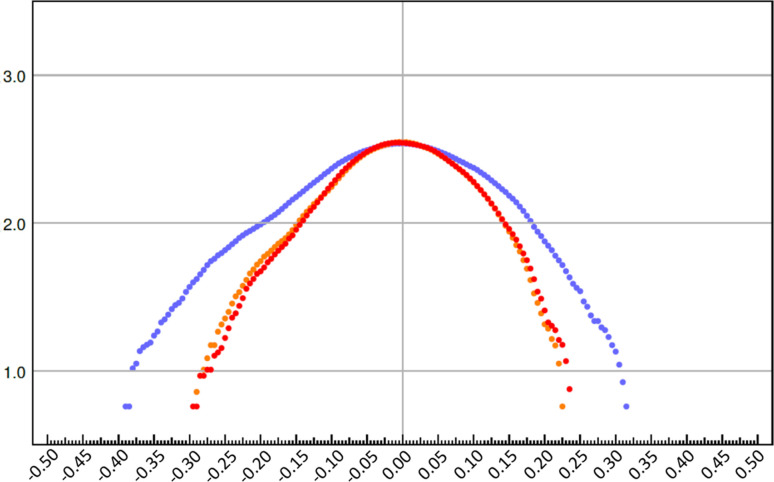
Fractal dimension (*y* axis) versus residual potential (eÅ^−1^) (*x* axis) for dynamical refinements on experimental data with various scattering models: IAM (blue), TAAM (red) and crystal-TAAM (orange). Values were calculated using the *jnk2RDA* program (Meindl & Henn, 2008 ▸).

The most noticeable improvement in the fractal plots (Fig. 14 ▸) is again visible when changing the model from IAM to TAAM. The shape of the curve is narrower and closer to the parabola. Comparing TAAM and crystal-TAAM, we see that the deviation from the parabola shape on the positive side is slightly smaller but the deviation on the negative side is slightly larger. However, both plots are similar, judging both by their shape and width.

#### 3.3.5. *X*—H bond lengths

**Figure 15:**
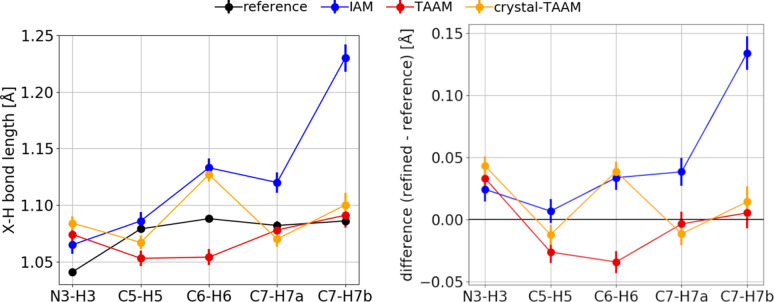
Left: *X*—H bond lengths (Å), error bars equal to the e.s.d. Right: difference (*X*—H_refinement_ − *X*—H_reference_), error bar calculated from error propagation. All data from dynamical refinements on experimental data. Reference: values from neutron data at 60 K.

The TAAM dynamical refinement results in average *X*—H bond lengths closer to the reference than IAM, with ME = 0.047 Å and RMSD = 0.065 Å for IAM and ME = −0.005 Å and RMSD = 0.025 Å for TAAM (Table 4 ▸, Fig. 15 ▸). Bond lengths from TAAM and crystal-TAAM are similar, only for C6—H6 there is a significant difference. IAM gives systematically too long bond lengths, whereas lengths from TAAM are sometimes too long and sometimes too short, but in general are better than those from IAM, judging by the lower ME and RMSD values. The observed trends are consistent with theoretical simulations. Despite the improvement, it can be concluded that obtaining accurate *X*—H distances is at the limit of what is possible with the presented experimental data.

#### 3.3.6. ADPs

**Figure 16:**
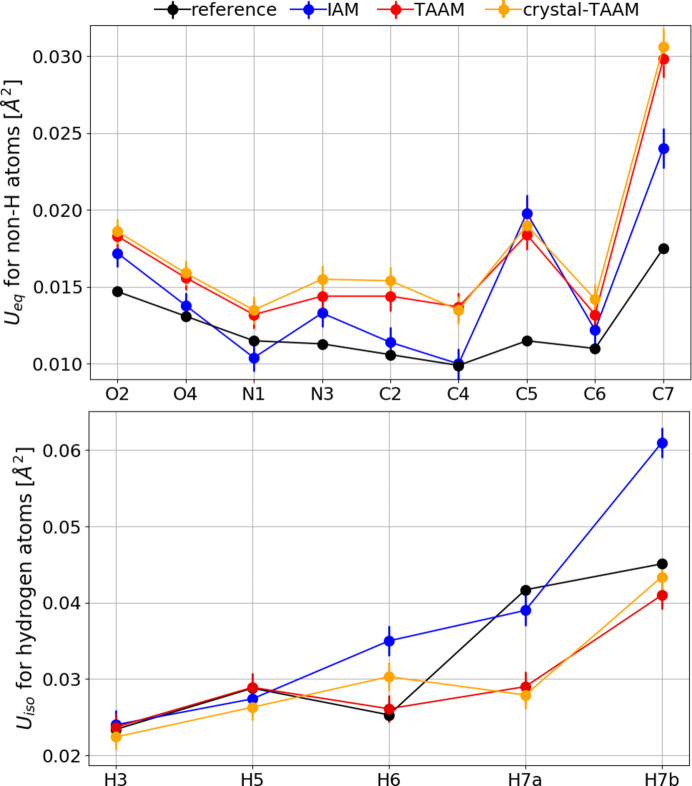
(Top) *U*_eq_ for non-hydrogen atoms. (Bottom) *U*_iso_ for hydrogen atoms (Å^2^) with uncertainties. All data from dynamical refinements on experimental data. Reference: values interpolated to 100 K from neutron data at 60 and 123 K.

Unlike in the case of bond lengths, we cannot directly compare the values of ADPs with a reference, as the neutron reference structure was measured at a different temperature. Furthermore, a small contribution of radiation damage cannot be excluded. However, it is possible to say that ADPs for non-hydrogen atoms from TAAM and crystal-TAAM dynamical refinements are larger than ADPs from IAM (Fig. 16 ▸, Table S8). The opposite trend is true for hydrogen atoms: the ADPs are on average smaller in the case of TAAM and crystal-TAAM than for IAM. The trends follow those observed for the simulated data.

#### 3.3.7. Crystal thickness

Crystal thickness is a refinable parameter specific for dynamical refinement. Note that its value also depends on the applied scattering model (see Table S9). Crystal thickness refined to a relatively large value of approximately 130–150 nm, and tends to grow by up to 7% on changing from IAM to TAAM. Experience with dynamical refinement shows that improved models tend to result in increased thickness. Thus, this observation also supports the conclusion that TAAM is a better fit to the experimental data.

## 4. Conclusions

Dynamical refinement provides a substantial improvement compared with the kinematic approach in all aspects (from the fitting of the model to the data and geometry). The improved fit allows us to observe the fine features of the electrostatic potential and, in the present case, it allowed us to observe the traces of deformation potential due to interatomic interactions. Several variants of TAAM refinements were performed to assess the improvement they offer over IAM. Standard TAAM with multipolar parameters extracted from the UBDB/MATTS database cleaned most of the residual potential on covalent bonds and around the atoms. However, surprisingly, the refinement figures of merit [*e.g.* *R*(*F*)] remained high, especially for low-angle reflections, and there were high residues in the areas of intermolecular interactions. The effect was clearly observed for both experimental and simulated data. The result improved when the multipolar parameters for the TAAM refinement were obtained, not from the database, but by fitting theoretical structure factors obtained by DFT calculations directly on the investigated structure (crystal-TAAM). This model yielded lower figures of merit and lower difference potentials in the hydrogen-bond region, the latter clearly visible only on simulated data. The third model tested, the refinement with multipolar parameters refined against DFT-calculated structure factors for a single isolated molecule (molecule-TAAM), provides worse results than standard TAAM. These results, particularly the differences between various TAAM versions, show that the electrostatic potential is very sensitive to intermolecular interactions. It seems more difficult to find a good TAAM for ED data than for X-ray diffraction, and optimizing the multipolar parameters for the specific structure using *ab initio* calculations appears necessary for obtaining significant improvement over IAM.

The TAAM dynamical refinements on experimental 3D ED data still present relatively high values of fitting statistics (*R* factors) compared with what is observed for X-ray diffraction, especially if the crystals exhibit significant imperfections. However, this work shows that the quality of 3D ED data and dynamical refinement is already sufficient to detect minute variations of the electrostatic potential due to bonding interactions and even the small variations caused by intermolecular interactions.

Raw diffraction images and associated data are available online at https://10.5281/zenodo.10079328. The CIFs and all refinements presented in this work are provided in the supporting information.

## Supplementary Material

Crystal structure: contains datablock(s) IAM, TAAM, crystalTAAM. DOI: 10.1107/S2052252524001763/of5004sup1.cif

Structure factors: contains datablock(s) IAM. DOI: 10.1107/S2052252524001763/of5004IAMsup2.hkl

Structure factors: contains datablock(s) TAAM. DOI: 10.1107/S2052252524001763/of5004TAAMsup3.hkl

Structure factors: contains datablock(s) crystalTAAM. DOI: 10.1107/S2052252524001763/of5004crystalTAAMsup4.hkl

Additional tables and figures. DOI: 10.1107/S2052252524001763/of5004sup5.pdf

CCDC references: 2334661, 2334662, 2334663

## Figures and Tables

**Table 1:** *X*—H bond lengths (Å) with uncertainties from kinematical refinements against simulated 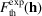 kinematical data Anisotropic ADPs were applied to all atoms. In the last two rows, the mean error {ME = ∑[(*X*—H_refinement_) − (*X*—H_reference_)]/*N*} and root-mean-square-deviation statistics (RMSD = {∑[(*X*—H_refinement_) − (*X*—H_reference_)]^2^}^1/2^/*N*) are given. Reference values were obtained from neutron data at 15 K.

Bond	Reference (15 K)	IAM	TAAM	Molecule-TAAM	Crystal-TAAM
N3—H3	1.0425	1.045 (4)	1.043 (2)	1.030 (3)	1.0467 (17)
C5—H5	1.0812	1.101 (6)	1.076 (2)	1.084 (4)	1.0727 (19)
C6—H6	1.0877	1.111 (5)	1.080 (2)	1.098 (4)	1.0790 (18)
C7—H7a	1.0826	1.102 (6)	1.078 (2)	1.092 (4)	1.074 (2)
C7—H7b	1.0878	1.113 (4)	1.0915 (19)	1.087 (3)	1.0989 (16)

ME	NA	0.018	−0.003	0.002	−0.002
RMSD	NA	0.020	0.005	0.008	0.009

**Table 2:** The mean value of 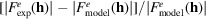 (%) for the first eight low-angle groups of symmetry-equivalent reflections from dynamical refinements on experimental data Data for crystals 1 and 2 are presented in the first and second rows. NA: the reflection is not available in the data from this crystal.

Symmetry-equivalent reflections	Resolution, *d* (Å)	No. of reflections	IAM (%)	TAAM (%)	Crystal-TAAM (%)
{110}	9.3	16	−16	−26	−8
		1	−11	−30	−8
{220}	4.7	16	14	19	21
		1	48	48	61
{130}	4.2	9	−29	4	15
		3	−28	16	26
{321}	3.1	0	NA	NA	NA
		2	−31	−17	−16
{231}	3.1	0	NA	NA	NA
		4	−33	−17	−27
{330}	3.1	17	−4	19	−7
		1	42	61	30
{200}	6.6	2	22	−1	36
		0	NA	NA	NA
{020}	6.6	3	−22	−27	−26
		0	NA	NA	NA

**Table 3:** R(*F*) statistics (%) for dynamical refinements on experimental data with various scattering models: IAM, TAAM and crystal-TAAM *R*_obs_(*F*): including reflections with *I*>3σ(*I*). *R*_all_(*F*): including all reflections.

	Crystal	No. of reflections	IAM (%)	TAAM (%)	Crystal-TAAM (%)
*R*_obs_(*F*)	1	2408	9.07	8.89	8.44
	2	3001	9.84	8.95	8.96
	Overall	5409	9.53	8.92	8.75
*R*_all_(*F*)	1	5220	12.62	12.15	11.74
	2	6361	14.19	13.12	13.13
	Overall	11581	13.55	12.73	12.57

**Table 4:** *X*—H bond lengths (Å) with uncertainties from dynamical refinements on experimental data Mean error: ME = ∑[(*X*—H_refinement_) − (*X*—H_reference_)]/*N*. Root-mean-square-deviation: RMSD = {∑[(*X*—H_refinement_) − (*X*—H_reference_)]^2^}^1/2^/*N*. NA: not applicable. Reference: values from neutron data at 60 K.

Bond	Reference (60 K)	IAM	TAAM	Crystal-TAAM
N3—H3	1.0408 (17)	1.065 (8)	1.074 (7)	1.084 (6)
C5—H5	1.0794 (19)	1.086 (8)	1.053 (7)	1.067 (6)
C6—H6	1.0884 (18)	1.122 (8)	1.054 (7)	1.127 (6)
C7—H7a	1.0817 (21)	1.120 (9)	1.078 (8)	1.070 (7)
C7—H7b	1.0858 (16)	1.220 (12)	1.091 (11)	1.100 (11)

ME	NA	0.047	−0.005	0.014
RMSD	NA	0.065	0.025	0.028
