# Supplementary material for: Dynamical refinement with multipolar electron scattering factors
Source: IUCrJ. 2024 Mar 21;11(Pt 3):309–24. doi: 10.1107/S2052252524001763 (PMC11067749; doi:10.1107/S2052252524001763)
Supplement: Supplementary file 5 [file m-11-00309-sup5.pdf]

IUCrJ

Volume 11 (2024)

Supporting information for article:

Dynamical refinement with multipolar electron scattering factors

Barbara Olech, Petr Brázda, Lukas Palatinus and Paulina Maria Dominiak

SUPPORTING INFORMATION

DYNAMICAL REFINEMENT WITH MULTIPOLAR ELECTRON SCATTERING FACTORS

Barbara Olech,[1#] Petr Brázda,[2] Lukas Palatinus,[2*] and Paulina Maria Dominiak[1*]

[1]Biological and Chemical Research Centre, Department of Chemistry, University of Warsaw, Warsaw (Poland),

[2]Institute of Physics of the Czech Academy of Sciences, Na Slovance 1999/2, 182 00 Prague , Czech Republic

[#]maiden name: Gruza

Table S-1. Local coordinates systems for HC-MM refinement of 1-methyluracil structure against $F^{x}_{molecule-static}(\boldsymbol{h})$ and $F^{x}_{crystal-static}(\boldsymbol{h})$ described in Chapter 2. First axis: orange, second axis: green.

	O(4)	O(2)	N(1)	N(3)	C(2)	C(4)	C(5)	C(6)	C(7)
x	C(4)	C(2)	DUM1	DUM1	DUM1	DUM1	DUM1	DUM1	H(7B)
y	N(3)	N(3)	C(6)	C(2)	N(3)	N(3)	C(6)	N(1)	
z									N(1)
symm	m	m	m	m	m	m	m	m	3m

	H(6)	H(5)	H(3)	H(7B)	H(7A)
x					
y	C(5)	C(6)	C(2)	N(1)	N(1)
z	C(6)	C(5)	N(3)	C(7)	C(7)
symm	cyl	cyl	cyl	cyl	cyl

DUM1 = (0.317483, 0.632873, 0.500000)

Table S-2. Plm and κ/ κ’ parameters from HC-MM refinement of 1-methyluracil structure against $F^{x}_{crystal-static}(\boldsymbol{h})$ described in the Section 2.4.

	O(4)	O(2)	N(1)	N(3)	C(2)	C(4)	C(5)
K0	0.9931(4)	0.9872(5)	1.0058(8)	1.0361(10)	1.0082(12)	1.0089(13)	1.052(2)
K’	0.997(7)	0.946(6)	0.973	1.033	0.908(5)	0.886(5)	0.930(10)
Pval	6.126(6)	6.198(8)	4.938(10)	4.586(16)	4.132(12)	4.174(14)	3.218(26)
P11	-0.0694(12)	-0.0814(13)	-0.0130(11)	0.0246(13)	-0.036(2)	-0.0023(18)	0.016(3)
P1-1	-0.0097(8)	-0.0137(8)	-0.0045(13)	-0.0137(11)	0.0163(16)	-0.0244(19)	0.016(3)
P10		0	0	0	0	0	0
P20	-0.0110(6)	-0.0229(7)	0.0385(8)	0.0294(8)	-0.1276(19)	-0.141(2)	-0.0379(18)
P21		0	0	0	0	0	0
P2-1		0	0	0	0	0	0
P22	-0.0423(8)	-0.0494(9)	-0.0010(10)	0.0027(10)	0.0501(18)	0.0461(17)	-0.0209(17)
P2-2	-0.0050(8)	-0.0070(8)	0.0038(10)	-0.0075(9)	-0.0119(15)	-0.0578(19)	0.027(2)
P30		0	0	0	0	0	0
P31	-0.0182(8)	-0.0200(8)	0.0148(10)	0.0051(11)	-0.0145(15)	0.0042(14)	0.0195(15)
P3-1	0.0010(8)	-0.0021(8)	0.0017(12)	0.0122(11)	0.0001(14)	0.0036(16)	0.0149(19)
P32		0	0	0	0	0	0
P3-2		0	0	0	0	0	0
P33	0.0198(10)	0.0203(9)	-0.0762(13)	-0.0458(13)	-0.170(3)	-0.183(3)	-0.097(4)
P3-3	-0.0032(9)	0.0015(8)	0.0060(14)	0.0162(14)	0.015(2)	0.020(2)	0.001(2)
P40	0.0100(9)	0.0073(10)	-0.0053(11)	0.0010(12)	0.0000(19)	-0.0000(18)	0.000(2)
P41		0	0	0	0	0	0
P4-1		0	0	0	0	0	0
P42	-0.0021(9)	-0.0134(12)	0.0000(11)	0.0021(13)	0.0258(18)	-0.0142(16)	-0.009(2)
P4-2	0.0032(11)	0.0070(11)	0.0027(15)	-0.0043(12)	0.0127(18)	0.007(2)	-0.0038(19)
P43		0	0	0	0	0	0
P4-3		0	0	0	0	0	0
P44	0.0126(10)	0.0083(12)	0.0211(14)	-0.0022(15)	-0.024(3)	-0.039(3)	0.057(3)
P4-4	0.0044(12)	0.0066(11)	0.0143(16)	0.0082(16)	-0.030(3)	0.008(3)	0.004(3)

	C(6)	C(7)	H(6)	H(5)	H(3)	H(7B)	H(7A)
K0	1.0278(17)	1.056(2)	0.953(4)	0.953(4)	0.939(6)	1.058(3)	1.058(3)
K’	0.805(4)	1.013(8)	1.019(6)	1.019(6)	1.228(13)	1.126(7)	1.126(7)
Pval	3.620(20)	3.236(26)	1.414(14)	1.442(14)	1.258(14)	1.205(8)	1.230(10)
P11	0.142(4)	0	0	0	0	0	0
P1-1	-0.060(4)	0	-0.011(3)	0.020(2)	-0.0037(11)	-0.0015(14)	0.0073(12)
P10	0	-0.0053(14)	0.197(4)	0.181(3)	0.123(3)	0.189(4)	0.113(2)
P20	-0.137(2)	-0.0389(14)	0.118(3)	0.081(3)	0.073(3)	0.080(3)	0.068(3)
P21	0	0	0	0	0	0	0
P2-1	0	0	-0.015(2)	-0.009(2)	-0.0013(18)	0.0010(16)	0.0133(18)
P22	-0.024(2)	0	0.006(3)	-0.021(2)	-0.0078(13)	-0.0064(15)	-0.0217(18)
P2-2	-0.061(3)	0	0	0	0	0	0
P30	0	0.100(2)					
P31	0.002(2)	0					
P3-1	-0.030(2)	0					
P32	0	0					
P3-2	0	0					
P33	-0.194(4)	0.082(2)					
P3-3	-0.008(3)	0					
P40	0.045(2)	0.0224(18)					
P41	0	0					
P4-1	0	0					
P42	-0.004(2)	0					
P4-2	-0.014(2)	0					
P43	0	-0.0406(19)					
P4-3	0	0					
P44	-0.027(4)	0					
P4-4	-0.020(4)	0					

Table S-3. Plm and κ/ κ' parameters from HC-MM refinement of 1-methyluracil structure against $F^{x}_{molecule-static}(\boldsymbol{h})$ described in the Section 2.4.

	O(4)	O(2)	N(1)	N(3)	C(2)	C(4)	C(5)
K0	0.98532(3)	0.98567(3)	0.99706(5)	0.99968(6)	0.99228(8)	0.98960(9)	1.01127(10)
K'	1.1223(5)	1.1403(6)	1.0652(7)	1.1254(9)	0.8450(3)	0.8501(3)	0.9694(7)
Pval	6.1654(11)	6.1642(11)	4.9893(12)	4.9337(17)	4.1198(15)	4.1211(16)	3.834(2)
P11	-0.1095(3)	-0.1074(3)	0.0015(2)	-0.0045(3)	-0.1127(5)	-0.0840(5)	0.0077(5)
P1-1	-0.00063(17)	0.00577(17)	-0.0101(2)	-0.0098(2)	0.0127(4)	-0.0393(5)	0.0154(4)
P10	0	0	0	0	0	0	0
P20	-0.06868(13)	-0.04523(12)	0.06494(19)	0.05669(17)	-0.2964(5)	-0.3039(6)	-0.0999(4)
P21	0	0	0	0	0	0	0
P2-1	0	0	0	0	0	0	0
P22	-0.09263(15)	-0.08556(16)	-0.00728(17)	-0.00579(14)	0.1159(4)	0.0968(4)	-0.0014(3)
P2-2	-0.00312(11)	-0.00015(11)	0.00660(16)	-0.01169(14)	0.0013(4)	-0.0813(4)	0.0162(3)
P30	0	0	0	0	0	0	0
P31	-0.00655(16)	-0.00431(16)	0.0139(2)	0.0104(2)	-0.0033(4)	-0.0030(4)	0.0319(3)
P3-1	0.00282(17)	0.00299(16)	0.0022(2)	-0.0008(2)	0.0041(4)	-0.0070(4)	0.0304(4)
P32	0	0	0	0	0	0	0
P3-2	0	0	0	0	0	0	0
P33	0.01166(16)	0.00922(16)	-0.1265(3)	-0.1042(3)	-0.4365(9)	-0.3936(9)	-0.2213(8)
P3-3	-0.00279(16)	-0.00107(16)	0.0044(2)	0.0062(2)	0.0194(5)	0.0521(5)	0.0141(4)
P40	-0.00527(18)	-0.00385(18)	0.0052(3)	0.0106(2)	0.0743(6)	0.0580(6)	0.0131(4)
P41	0	0	0	0	0	0	0
P4-1	0	0	0	0	0	0	0
P42	0.00277(17)	0.00119(17)	-0.0039(2)	-0.0004(2)	-0.0140(5)	-0.0204(5)	-0.0184(4)
P4-2	-0.00076(17)	-0.00087(17)	0.0008(2)	0.0010(2)	-0.0002(6)	-0.0065(6)	0.0149(4)
P43	0	0	0	0	0	0	0
P4-3	0	0	0	0	0	0	0
P44	0.00753(16)	0.00960(16)	0.0061(2)	0.0099(2)	-0.0791(6)	-0.0641(6)	0.0144(5)
P4-4	0.00001(16)	-0.00082(16)	0.0035(3)	-0.0023(2)	-0.0186(7)	-0.0583(6)	0.0006(5)

	C(6)	C(7)	H(6)	H(5)	H(3)	H(7B)	H(7A)
K0	1.00191(9)	1.01564(11)	1.1175(3)	1.1175(3)	1.1343(5)	1.1111(3)	1.1111(3)
K'	0.8901(4)	0.9245(4)	1.1480(8)	1.1480(8)	1.3996(15)	1.1331(6)	1.1331(6)
Pval	3.968(2)	3.661(3)	1.0157(12)	1.0069(12)	0.9072(13)	1.0333(10)	1.0478(11)
P11	0.0653(6)	0					
P1-1	-0.0846(5)	0					
P10	0	-0.0316(4)	0.1917(7)	0.1746(6)	0.1475(6)	0.1876(5)	0.1832(5)
P20	-0.2086(5)	-0.0944(3)	0.0959(6)	0.0710(6)	0.0631(5)	0.0856(5)	0.0853(5)
P21	0	0					
P2-1	0	0					
P22	-0.0230(4)	0					
P2-2	-0.0841(4)	0					
P30	0	0.2715(6)					
P31	0.0211(4)	0					
P3-1	-0.0482(4)	0					
P32	0	0					
P3-2	0	0					
P33	-0.3017(8)	0.2183(5)					
P3-3	0.0282(4)	0					
P40	0.0318(5)	0.0804(5)					
P41	0	0					
P4-1	0	0					
P42	-0.0142(5)	0					
P4-2	-0.0224(5)	0					
P43	0	-0.0838(5)					
P4-3	0	0					
P44	-0.0114(5)	0					
P4-4	-0.0520(5)	0					

Table S-4. F^{e}_{model} values in [Å] calculated for the target geometry of 1-methyluracil.

Reflections Family	$\|F_{th}(h)\|$	$\|F_{IAM}(h)\|$	$\|F_{TAAM}(h)\|$	$\|F_{crystal\text{-}TAAM}(h)\|$	$\|F_{molecule\text{-}TAAM}(h)\|$
{110}	14.068084	15.721748	17.577727	13.567705	7.546553
{020}	28.831406	31.995038	26.472771	28.748049	24.758199
{200}	4.306391	2.087430	6.258119	2.611364	2.966456
{220}	6.536436	7.846348	6.552135	6.676652	6.265080
{121}	12.621292	13.614458	13.057293	12.669249	13.051564
{211}	42.8667	44.475621	42.246443	42.91239	42.274023
{130}	3.935353	7.571214	4.450185	4.136944	3.931630
{310}	3.230480	4.773987	2.841924	2.791781	3.560066
{040}	8.853079	7.412448	8.475894	8.658254	8.286815
{400}	8.042823	9.124095	8.495320	8.185125	8.791650
{231}	13.856190	16.665160	13.165099	14.185904	13.770575
{321}	9.196195	12.006652	9.309780	9.113509	9.110989
{330}	18.425987	20.216793	18.117460	18.452740	17.881885
{002}	115.314483	127.687504	115.069379	114.629295	115.268022

Table S-5. $(|F^{e}_{th}(\boldsymbol{h})| - |F^{e}_{model}(\boldsymbol{h})|)/|F^{e}_{model}(\boldsymbol{h})|$ [%] for the low-angle reflections from models applied to the target crystal structure. All $|F^{e}(\boldsymbol{h})|$ were computed using the target atomic positions and thermal parameters. Absolute values of $|F^{e}_{th}(\boldsymbol{h})|$ for simulated theoretical data are given for the reference. Bold – reflections present in experimental data.

Symmetry equivalent reflections	$\|F^{e}_{th}(\mathbf{h})\|$ [Å]	IAM [%]	TAAM [%]	molecule- TAAM [%]	crystal- TAAM [%]
{110}	**14.1**	**-12**	**-25**	**46**	**4**
**{020}**	**28.8**	**-11**	**8**	**14**	**0**
**{200}**	**4.3**	**52**	**-45**	**31**	**39**
{220}	**6.5**	**-20**	**0**	**4**	**-2**
{121}	12.6	-8	-3	-3	0
**{211}**	**42.9**	**-4**	**1**	**1**	**0**
**{130}**	**3.9**	**-92**	**-13**	**0**	**-5**
**{310}**	**3.2**	**-48**	**12**	**-10**	**14**
**{040}**	**8.9**	**16**	**4**	**6**	**2**
**{400}**	**8.0**	**-13**	**-6**	**-9**	**-2**
**{231}**	**13.9**	**-20**	**5**	**1**	**-2**
{321}	**9.2**	**-31**	**-1**	**1**	**1**
{330}	**18.4**	**-10**	**2**	**3**	**0**
{002}	115.3	-11	0	0	1

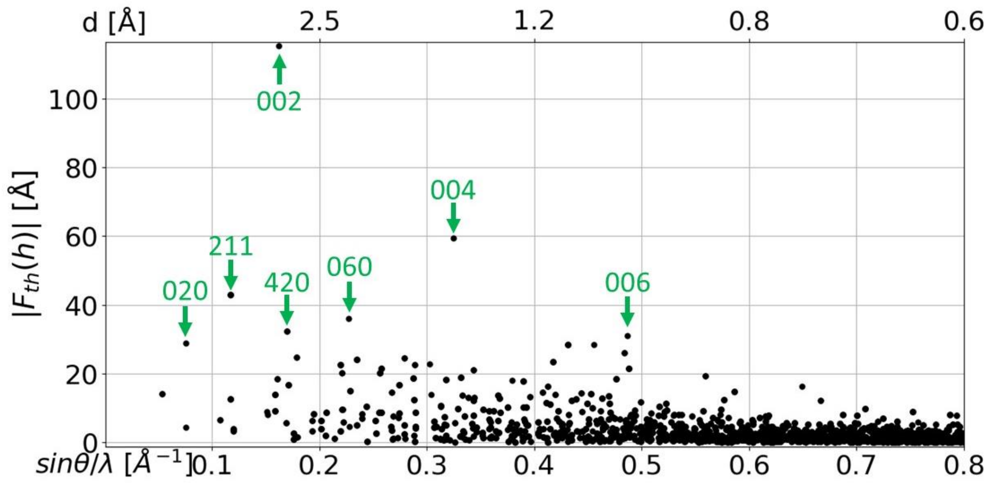

Figure S-1. $|F^{e}_{th}(\mathbf{h})|$ versus resolution (sinΘ/λ [$Å^{-1}$], d [Å]) calculated by periodic DFT for the target crystal structure of 1-methyluracil.

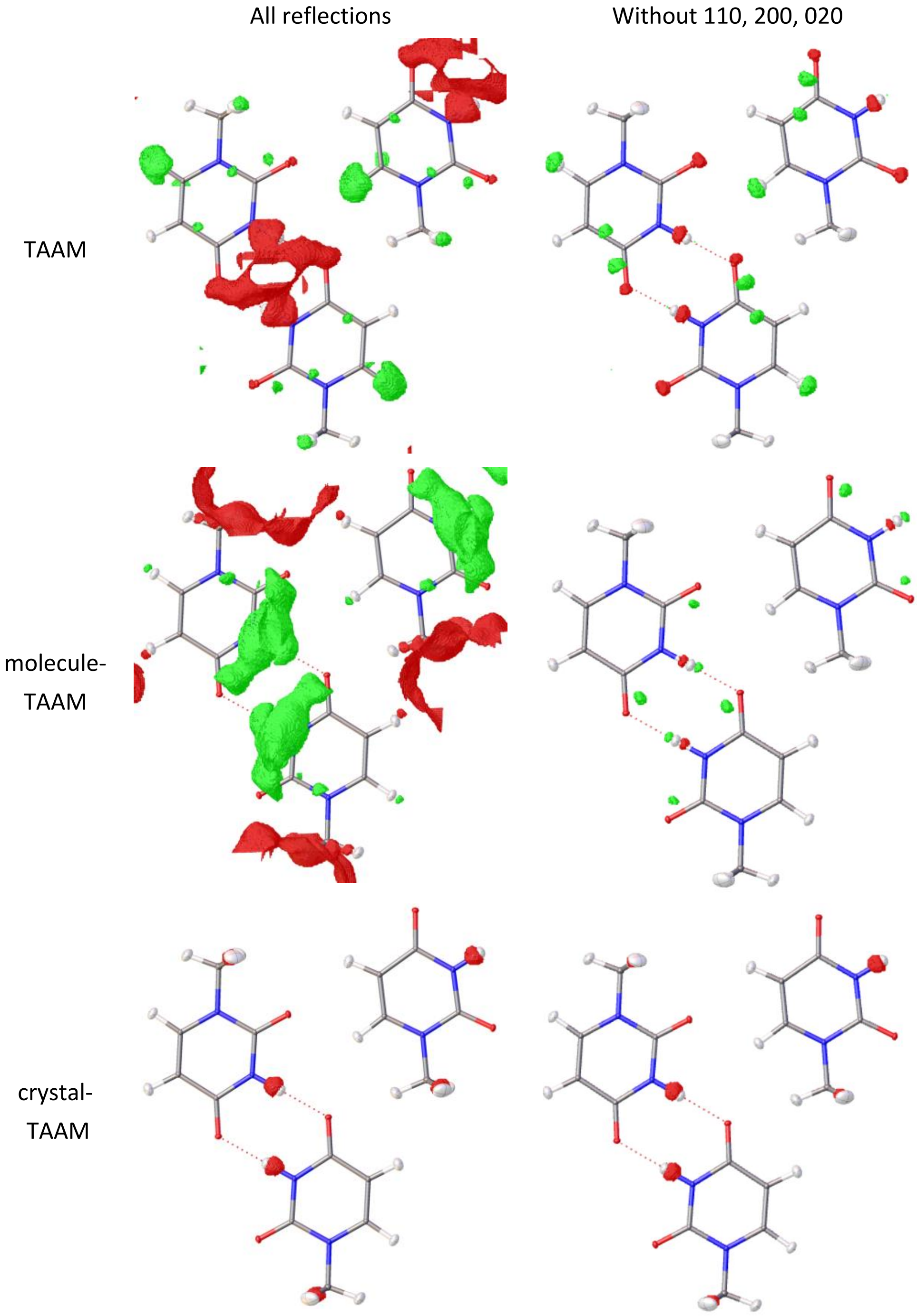

Figure S-2. Residual potential maps $[|F^{e}_{th}(\boldsymbol{h})| - |F^{e}_{model}(\boldsymbol{h})|]e^{i\varphi_{model}}$, for d_{min} = 0.60 Å, for the TAAM, molecule-TAAM' and crystal-TAAM models applied to the target crystal structure, calculated with Olex2-1.3. Contour level: 0.07 eÅ^{-1}. Green contours for positive potential and red for negative. Thermal ellipsoids with 50 % probability.

Table S-6. $(|F_{th}^{e}(\boldsymbol{h})| - |F_{model}^{e}(\boldsymbol{h})|)/|F_{model}^{e}(\boldsymbol{h})|$ [%] for the low-angle reflections from kinematical refinements on simulated $F_{th}^{e}(\boldsymbol{h})$ kinematical data. All $|F^{e}(\boldsymbol{h})|$ were computed by olex2-1.3. Absolute values of $|F_{th}^{e}(\boldsymbol{h})|$ for simulated theoretical data are given for the reference. Bold – reflections present in experimental data.

Symmetry-equivalent reflection	$\lvert F_{th}^{e}(\mathbf{h})\rvert$ [A]	IAM [%]	TAAM [%]	molecule-TAAM [%]	crystal-TAAM [%]
{110}	**14.1**	**-7**	**-14**	**45**	**4**
**{020}**	**28.8**	**-6**	**12**	**14**	**0**
**{200}**	**4.3**	**48**	**-37**	**25**	**31**
{220}	**6.5**	**-16**	**1**	**4**	**-3**
{121}	12.6	-4	-3	-4	-1
**{211}**	**42.9**	**1**	**2**	**1**	**0**
**{130}**	**3.9**	**-81**	**-9**	**-1**	**-5**
**{310}**	**3.2**	**-49**	**7**	**-15**	**10**
**{040}**	**8.9**	**14**	**5**	**5**	**2**
**{400}**	**8.0**	**-7**	**-3**	**-9**	**0**
{231}	**13.9**	**-14**	**6**	**1**	**-2**
{321}	**9.2**	**-24**	**-1**	**1**	**1**
{330}	**18.4**	**-5**	**3**	**3**	**-1**
{002}	115.3	-6	1	0	0

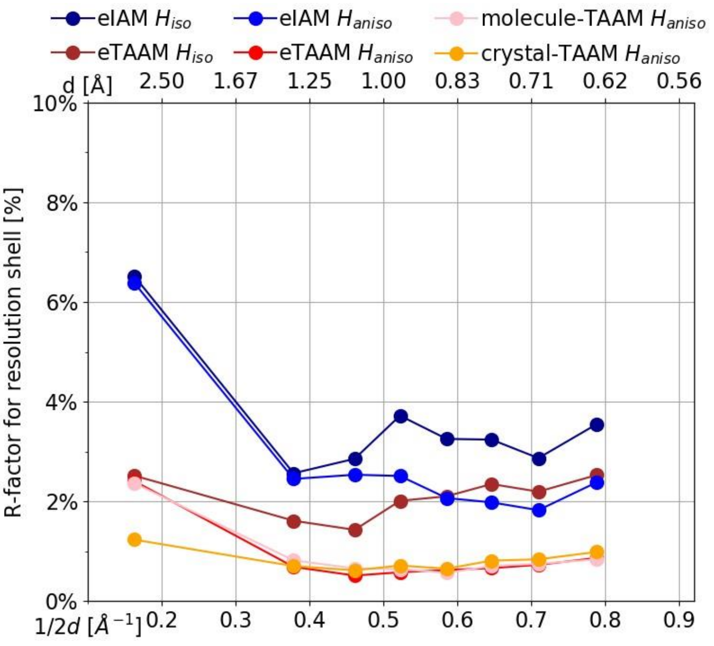

Figure S-3. Mean $R_{all}(F)$ [%] for eight resolution shells calculated from kinematical refinements on simulated $F_{th}^{e}(\boldsymbol{h})$ kinematical data.

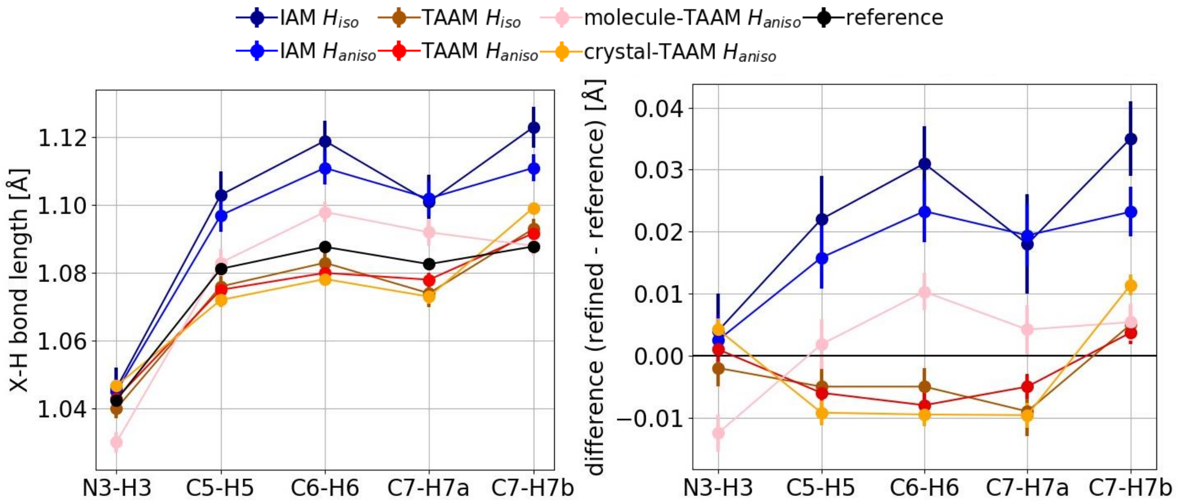

Figure S-4. On the left: X–H bond lengths [Å], error bar equal to one esd. On the right: difference (X–$H_{refinement}$ - X–$H_{reference}$), error bar calculated from error propagation. All data from kinematical refinements against simulated $F_{th}^{e}(\boldsymbol{h})$ kinematical data. Anisotropic ADPs were applied to all atoms. Reference – target values from neutron data at 15 K.

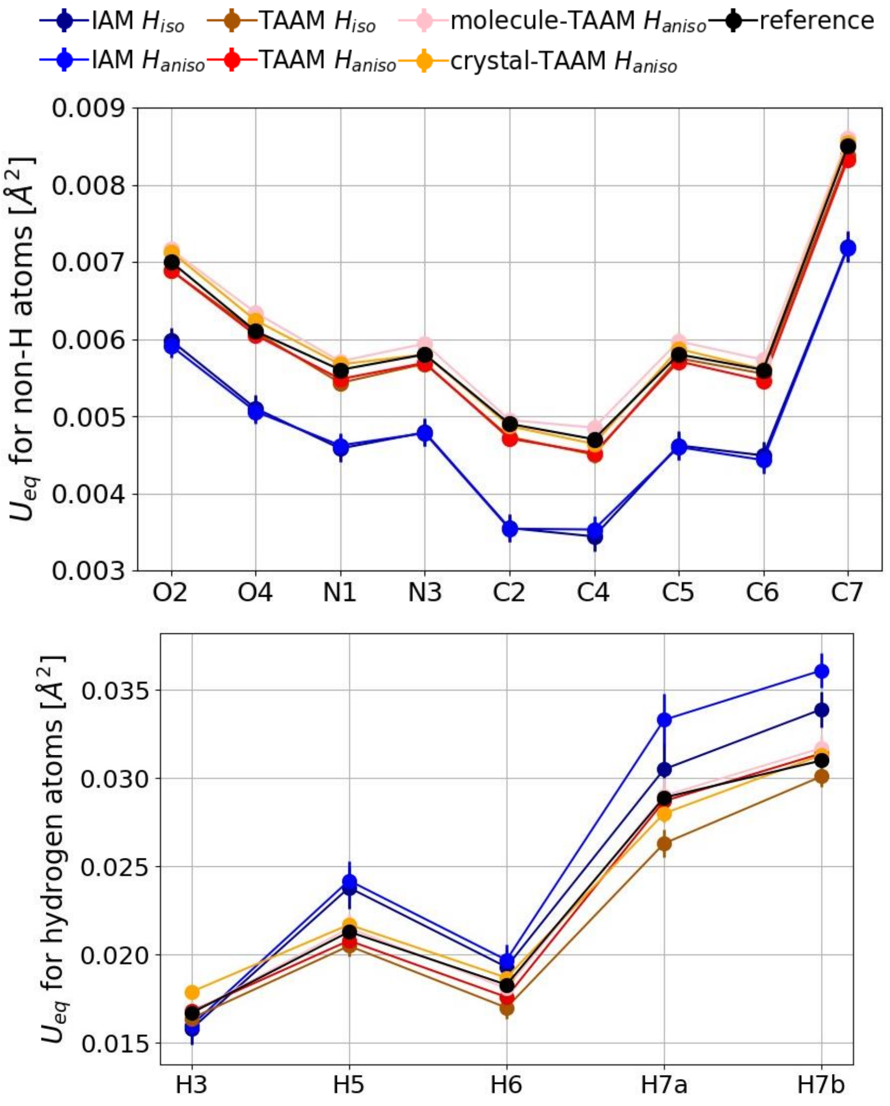

Figure S-5. U_{eq} for non-hydrogen atoms (upper) and U_{eq} for hydrogen atoms (lower) [$Å^2$] with uncertainties. All data for from kinematical refinements against simulated $F_{th}^{e}(\boldsymbol{h})$ kinematical data.

Table S-7. U_{eq} [Å^2] atoms with uncertainties. All data for from kinematical refinements against simulated $F^e_{th}(\boldsymbol{h})$ kinematical data. Anisotropic ADPs were applied to all atoms. Reference – target values from neutron data at 15 K. In the last four rows, mean error (ME=($U_{refienement}$)-(U_{model})/N) and root-mean-square-deviation (RMSD) statistics.

Atom	Reference	IAM	TAAM	molecule-TAAM	crystal-TAAM
O2	0.0070	0.00591(15)	0.00689(6)	0.00716(10)	0.00713(5)
O4	0.0061	0.00506(16)	0.00605(6)	0.00634(10)	0.00624(5)
N1	0.0056	0.00462(16)	0.00548(6)	0.00571(10)	0.00567(5)
N3	0.0058	0.00478(16)	0.00569(6)	0.00594(11)	0.00580(6)
C2	0.0049	0.00354(17)	0.00471(7)	0.00495(11)	0.00487(6)
C4	0.0047	0.00353(17)	0.00452(7)	0.00485(12)	0.00464(6)
C5	0.0058	0.00460(17)	0.00571(7)	0.00597(12)	0.00587(6)
C6	0.0056	0.00443(17)	0.00546(7)	0.00573(12)	0.00561(6)
C7	0.0085	0.00718(18)	0.00833(8)	0.00860(13)	0.00855(7)
H3	0.0167	0.0160(8)	0.0168(4)	0.0167(7)	0.0179(4)
H5	0.0213	0.0242(11)	0.0208(5)	0.0215(8)	0.0217(4)
H6	0.0183	0.0197(9)	0.0176(4)	0.0181(7)	0.0187(4)
H7a	0.0289	0.0333(15)	0.0287(6)	0.0290(10)	0.0280(5)
H7b	0.0310	0.0361(10)	0.0314(4)	0.0317(7)	0.0313(4)
ME non-H	NA	-0.00115	-0.00013	0.00014	0.00004
ME H	NA	0.0026	-0.0002	0.0002	0.0003
RMSD non-H	NA	0.00116	0.00014	0.00015	0.00008
RMSD H	NA	0.0034	0.0004	0.0003	0.0007

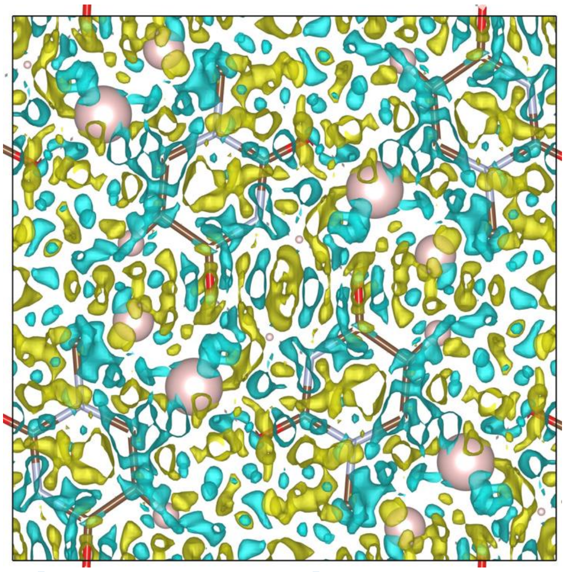

Figure S-6. Residual potential map $\left[\left|F^e_{exp}(\boldsymbol{h})\right| - \left|F^e_{IAM}(\boldsymbol{h})\right|\right]e^{i\varphi_{IAM}}$, 0.28 · 10^{-2} eÅ^{-1} contour level, cyan: negative, yellow: positive. Thermal ellipsoids with 50 % probability.

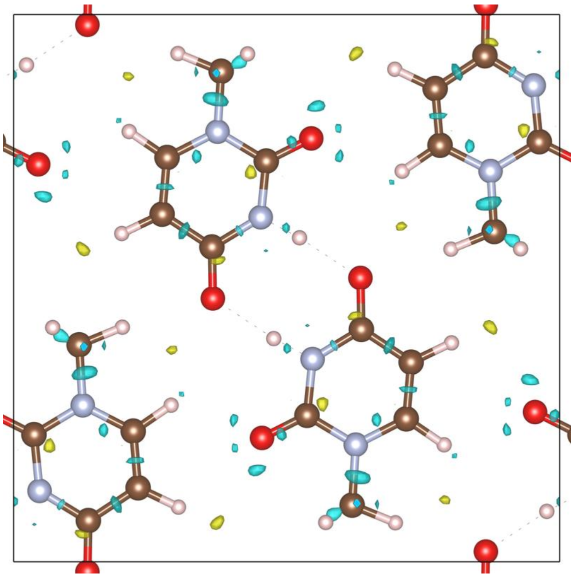

Figure S-7. Residual potential maps $[|F_{exp}^{e}(\boldsymbol{h})| - |F_{IAM}^{e}(\boldsymbol{h})|]e^{i\varphi_{IAM}}$ maps. 0.23 eÅ^{-1} contour level, cyan: negative, yellow: positive. Thermal ellipsoids with 50 % probability.

Table S-8. U_{eq} [Å^2] for non-hydrogen and U_{iso} [Å^2] for hydrogen atoms with uncertainties from dynamical refinements on experimental data. In the last four rows, mean error (ME) and root-mean-square-deviation (RMSD) statistics. NA – not applicable. Reference – values interpolated to 100 K from neutron data at 60 K and 123 K.

Atom	Reference	IAM	TAAM	crystal-TAAM
O2	0.0147	0.0172(9)	0.0183(8)	0.0186(8)
O4	0.0131	0.0138(8)	0.0156(8)	0.0159(8)
N1	0.0115	0.0104(9)	0.0132(9)	0.0135(9)
N3	0.0113	0.0133(9)	0.0144(9)	0.0155(9)
C2	0.0106	0.0114(10)	0.0144(10)	0.0154(9)
C4	0.0099	0.0100(10)	0.0137(9)	0.0135(9)
C5	0.0115	0.0198(12)	0.0184(10)	0.0190(10)
C6	0.0110	0.0122(10)	0.0132(10)	0.0142(10)
C7	0.0175	0.0240(13)	0.0298(12)	0.0306(12)
H3	0.0234	0.0240(18)	0.0237(17)	0.0224(16)
H5	0.0288	0.0274(19)	0.0289(19)	0.0263(17)
H6	0.0253	0.035(2)	0.0261(18)	0.0303(19)
H7a	0.0417	0.039(2)	0.029(2)	0.0279(18)
H7b	0.0451	0.061(2)	0.0410(19)	0.0434(18)
ME non-H	NA	0.0023	0.0044	0.0050
RMSD non-H	NA	0.0037	0.0054	0.0060
ME H	NA	0.004	-0.003	-0.003
RMSD H	NA	0.008	0.006	0.007

Table S-9. Scale-like parameters refined during dynamical refinement against $F_{exp}^{e}(\boldsymbol{h})$ of 1-methyluracil (scale factors and crystal thickness).

	IAM	TAAM	crystal-TAAM
scale factors	1191.193	1204.886	1207.670
	1250.425	1266.473	1267.358
	1299.109	1319.221	1322.145
	1321.759	1341.887	1347.209
	1342.539	1363.165	1365.698
	1337.109	1354.089	1358.375
	1322.186	1337.564	1339.485
	1297.346	1328.390	1331.993
	1249.481	1308.158	1310.188
	1232.922	1278.552	1283.138
	1188.185	1192.958	1200.534
	1175.061	1181.725	1198.327
	1217.040	1233.701	1235.954
	1254.389	1286.617	1277.781
	1189.646	1210.885	1223.172
	1185.157	1176.374	1236.816
	1112.098	1111.546	1164.189
	1127.605	1112.868	1176.213
	1116.875	1103.479	1171.009
	1116.430	1116.650	1170.871
	1134.474	1112.653	1194.615
	1114.518	1104.982	1145.197
	1149.224	1158.712	1161.712
	1115.327	1106.476	1132.757
	1111.351	1110.357	1113.529
	1075.629	1075.415	1079.624
	1571.211	1584.405	1582.505
	1540.141	1541.458	1548.560
	1612.258	1617.380	1624.555
	1553.198	1567.996	1565.367
	1496.024	1511.449	1495.653
	1504.730	1499.315	1502.557
	1463.481	1474.633	1474.612
	1409.363	1451.162	1455.233
	1327.496	1371.661	1383.380
	1346.196	1367.323	1371.128
	1319.851	1353.074	1350.248
	1258.957	1328.690	1314.841
	1355.309	1353.764	1353.965
	1350.053	1345.171	1346.655
	1350.606	1387.351	1390.684
	1309.815	1338.913	1336.471
	1258.139	1293.850	1294.679
	1282.910	1311.917	1315.287
	1237.710	1275.365	1279.021
	1278.333	1318.825	1322.415
	1243.950	1277.573	1279.768
	1455.580	1532.263	1525.676
	1430.977	1498.298	1490.800
	1455.054	1497.080	1506.493
	1358.528	1419.630	1421.979
	1349.688	1451.346	1459.521
	1421.620	1479.979	1476.605
	1502.849	1535.932	1534.727

	1455.378	1492.804	1492.413
	1535.225	1577.183	1576.582
	1464.082	1503.872	1506.617
	1665.541	1699.306	1705.761
	1572.829	1614.497	1617.663
	1602.786	1655.131	1654.943
	1600.225	1648.665	1650.320
	1642.636	1686.154	1674.963
	1611.542	1647.435	1637.449
	2076.169	2135.043	2134.880
	2125.667	2165.585	2179.542
	2073.329	2125.474	2124.963
	2089.063	2150.950	2149.609
	2085.703	2159.871	2154.987
	2088.060	2147.713	2152.328
	2065.375	2125.671	2122.249
	2150.654	2219.182	2215.583
	2163.216	2232.665	2224.557
	2062.730	2133.076	2139.568
	2055.959	2126.563	2128.153
	1957.867	2015.305	2019.568
	1905.534	1966.516	1966.721
	1748.574	1828.950	1835.105
	1701.550	1774.801	1777.849
	1497.551	1551.062	1571.702
	2075.824	2143.452	2151.763
	2064.214	2134.831	2139.780
	2053.859	2106.941	2105.685
	2001.286	2063.853	2071.834
	1692.052	1758.571	1749.328
	1608.249	1654.628	1668.382
	1646.392	1668.908	1680.509
	1523.162	1609.816	1596.684
	1541.451	1588.561	1595.138
	1491.818	1538.959	1537.518
	1484.930	1529.674	1533.880
	1470.972	1510.230	1512.519
	1449.846	1485.249	1488.369
	1388.493	1425.162	1424.793
	1342.791	1382.324	1379.110
Mean scale	1283.384	1296.632	1309.620
	1662.377	1714.870	1716.361
%	100	101.0	102.0
	100	103.2	103.2
thickness	1272.188	1362.300	1406.442
parameters	1411.141	1443.379	1496.966
%	100	107.1	110.6
	100	102.3	106.1
